# A novel 3’tRNA-derived fragment tRF-Val promotes proliferation and inhibits apoptosis by targeting EEF1A1 in gastric cancer

**DOI:** 10.1038/s41419-022-04930-6

**Published:** 2022-05-18

**Authors:** Huaiping Cui, Han Li, Hao Wu, Fengying Du, Xiaozhou Xie, Shujie Zeng, Zihao Zhang, Kangdi Dong, Liang Shang, Changqing Jing, Leping Li

**Affiliations:** 1 Department of Gastrointestinal Surgery, Shandong Provincial Hospital, Cheeloo College of Medicine, Shandong University, 250021 Jinan, Shandong China; 2 Department of Gastrointestinal Surgery, Shandong Provincial Hospital Affiliated to Shandong First Medical University, 250021 Jinan, Shandong China; 3 Shandong Provincial Laboratory of Translational Medicine Engineering for Digestive Tumors, Shandong Provincial Hospital, 250021 Jinan, Shandong China; 4 Department of Gastrointestinal Surgery, the First Affiliated Hospital of Shandong First Medical University, Shandong Provincial Qianfoshan Hospital, 250013 Jinan, Shandong China

**Keywords:** Cancer genomics, Small RNAs

## Abstract

At present, it is commonly believed that tRFs and tiRNAs are formed by the specific and selective shear of tRNAs under certain pressure stimulation, rather than by random degradation of tRNA. tRFs and tiRNAs have been reported to contribute to the biological process of a variety of human cancers. However, the evidence for the mechanisms of tRFs and tiRNAs in the occurrence and development of gastric cancer (GC) is still insufficient. Here, we aimed to explore the carcinogenic roles of tRFs and tiRNAs in GC with RNA-sequencing technique, and found a novel 3’tRNA-derived fragment tRF-Val was significantly upregulated in GC tissues and cell lines. tRF-Val expression was positively correlated with tumor size and the depth of tumor invasion in GC tissues. Functionally, tRF-Val promoted proliferation and invasion, and inhibited apoptosis in GC cells. Mechanistically, tRF-Val directly bound to the chaperone molecule EEF1A1, mediated its transport into the nucleus and promoted its interaction with MDM2 (a specific p53 E3 ubiquitin ligase), thus inhibiting the downstream molecular pathway of p53 and promoting GC progression. These findings provided a new potential therapeutic target for GC and a new explanation for the occurrence of GC.

## Introduction

As the fourth leading cause of cancer death, gastric cancer (GC) poses a serious threat to people’s health all over the world, especially in East Asia [1]. Research on the molecular mechanism of carcinogenesis and metastasis of GC has always been a hot topic [2]. It has been found that a variety of biomolecules are involved in the progress of GC, including coding genes [3–5], long non-coding RNAs [6–8], miRNAs [9–11], pseudogenes [12–14], and so on, and they construct a complex molecular regulatory network of GC.

In recent years, profiting from the development of high-throughput technology and microarray technology, people have been exploring new types of tumor targets, and one of them, a tRNA-derived small non-coding RNA (tRFs and tiRNAs), has gradually become a new focus of tumor research [15, 16]. tiRNAs contains 5’tiRNAs and 3’tRNAs, which were generated from the halves of tRNAs specific cleavage. tRNA related fragments (tRFs) are divided into five special types, tRF-1, tRF-2, tRF-3, and tRF-5 according to their different locations. Several studies have clarified that tRFs and tiRNAs are widely involved in the progression of various cancers by directly targeting binding RNAs or proteins. For example, it was reported that a 5’-tRNA halve, tiRNA-Gly could promote progression in papillary thyroid cancer by binding to RBM17 protein [17]; and a novel 3’-tRNA-derived fragment tRF3E could suppress the progression of breast cancer by targeting nucleolin [18]; besides, tRF03357 was found to promote progression in ovarian cancer by downregulating HMBOX1 [19]. Although tRFs has been deeply studied in some solid tumors, it is still rarely studied in GC.

In this study, with RNA-sequencing technique, we tried to explore the carcinogenic roles of tRFs and tiRNAs in GC by detecting their expression in four pairs of tumor tissues and paired normal tissues. The result showed that tRF-3a was the main upregulated type, while tRF-5a and tRF-5c were the main downregulated types in GC, indicating that tRF-3a may play a more important role in the occurrence and progression of gastric cancer. In the subsequent qPCR validation, a tRF-3a type tRF-60:76-Val-CAC-2 (hereinafter referred to as tRF-Val) was found to be most significantly upregulated in GC tissues, and increasing tRF-Val positively correlated with tumor size and the depth of tumor invasion. Moreover, we discovered that tRF-Val promoted proliferation and invasion, and inhibited apoptosis in GC cells. Mechanistically, we identified that tRF-Val directly bound to the chaperone molecule EEF1A1, mediated its transport into the nucleus, and promoted its interaction with MDM2 (a specific p53 E3 ubiquitin ligase), thus inhibiting the downstream molecular pathway of p53 and promoting GC progression. These findings provide new molecular mechanisms and therapeutic targets for the occurrence and progress of GC.

## Materials and methods

### Patient tissue samples

All GC tissues and control normal tissues were collected from patients who underwent gastrectomy in Shandong Provincial Hospital between 2015 and 2019 and preserved in liquid nitrogen. All patients had a definite preoperative and postoperative pathological diagnosis of gastric adenocarcinoma. All specimens were collected with the informed consent of the patients. The study was performed in accordance with the Declaration of Helsinki and approved by the Committee for Ethical Review of Research involving Human Subjects of Shandong Provincial Hospital.

### Cell lines

The human GC cell lines AGS, HGC-27, MKN-45, MKN-28, and immortalized human gastric epithelium cell line GES-1 were purchased from the Culture Collection of Chinese Academy of Sciences (Shanghai, China). All the cell lines were clearly verified by short tandem repeat (STR) analysis and eliminated mycoplasma contamination. All cells were cultured in RPMI-1640 complete medium (KeyGEN, Nanjing, China), containing 10% fetal bovine serum (FBS, Gibco, NY, USA) and 1% penicillin-streptomycin (Gibco). All cells were cultured in the 5% CO_2_ incubator at 37 °C.

### The tRFs and tiRNAs sequencing and data analysis

Four GC tissues and paired normal tissues were collected for tRFs and tiRNAs sequencing. The sequencing libraries were absolutely quantified using Agilent BioAnalyzer 2100. The sequencing analysis was performed via the Illumina NextSeq 500 platform (Aksomics, Shanghai, China) according to the manufacturer’s protocol.

### Quantitative real-time polymerase chain reaction (qRT-PCR)

The total RNAs of human GC tissues and GC cell lines were extracted using Trizol reagent (Takara, Japan). The Evo M-MLV RT Premix (Accurate Biotechnology, Hunan, China) was used for mRNA reverse transcription in a 10 μl reaction volume according to the manufacturer’s protocol. The miRNA 1st Strand cDNA Synthesis Kit (Accurate) was used for tRFs reverse transcription in a 10 μl reaction volume and then diluted to 100 μl. The cDNA amplification was performed by qRT-PCR using SYBR Green Pro Taq HS Premix (Accurate) with the Light Cycler 480 detection system (Roche Diagnostics, Basel, Switzerland). β-actin was used as the internal control for mRNA, and U6 was used as the internal control for tRFs. The expressions of genes related to internal controls were detected using the 2^−ΔΔCt^ method, and each assay was repeated three times. The sequence information of all primers was listed in Supplementary Table 1.

### Cell transfection

The tRF-Val overexpression model (oe-Val) and the control (oe-NC) were constructed by the transfection with the tRF-Val mimics and the negative control oligonucleotide designed and synthesized by Genomeditech (Shanghai, China). The tRF-Val knockdown model (sh-Val) and the control (sh-NC) were constructed by the transfection with the tRF-Val knockdown lentivirus and the negative control lentivirus designed and synthesized by Genomeditech. Small interfering RNAs against EEF1A1 (si-EEF1A1) and the negative control (si-NC), and the plasmid of EEF1A1 (oe-EEF1A1) and the negative control (vector) were designed and synthesized by Genomeditech. Lipofectamine 3000 reagent (Invitrogen, CA, USA) was used as the transfection aid reagent according to the manufacturer’s protocol. All the interfering sequences were listed in Supplementary Table 2.

### Cell counting kit-8 (CCK-8) proliferation assay

Transfected cells were seeded in 96-well plates with a density of 3000 cells per well. After the incubation of 0, 24, 48, 72, 96 h, 10 μl CCK-8 reagent (Kumanoto, Japan) was added to each well and incubated at 37 °C for 2 h. The optional density (OD) of each well at 450 nm was detected by the multifunctional microplate reader (Thermo Fisher Scientific, MA, USA). Each assay was repeated three times.

### Colony formation assay

Transfected cells were seeded in six-well plates with a density of 500–800 cells per well and cultured in a 5% incubator at 37 °C for 2 weeks. The medium of each well was sucked and discarded, and each well was washed with PBS for three times, fixed with paraformaldehyde for 30 min, and stained with crystal violet solution for 30 min. Finally, each well was photographed and the number of colonies was counted by the ImageJ software (NIH, Bethesda, Maryland, USA). Each assay was repeated three times.

### Flow cytometry apoptosis detection

Apoptotic cells were detected using the PE Annexin V Apoptosis Detection Kit (BD, NJ, USA) according to the manufacturer’s protocol. Annexin V-PE and 7-AAD were used as fluorescent identification dyes for apoptotic cells. Flow cytometry was used to detect the apoptosis rate with the help of technicians in the Central Laboratory of Shandong Provincial Hospital.

### Transwell invasion assay

The invasion assays were performed using the transwell chambers (Corning, NY, USA) with 8 μm-pore polycarbonate membranes. The transwell membranes were paved with matrigel mix (3 mg/ml) (BD, NJ, USA) for 1 h at 37 °C. The transfected cells (5 × 10^4^) were suspended in 200 ul serum-free medium and seeded in the upper chambers, and a medium containing 10% FBS without cells was added to the lower chamber as a chemoattractant. The transwell chambers were cultured in a 5% incubator at 37 °C for 24 h. Then the cells on the membrane of the upper chamber were wiped with cotton swabs, and the cells on the membrane of the upper chamber were washed with PBS for three times, fixed with paraformaldehyde for 30 min, and stained with crystal violet solution for 30 min. Finally, the chambers were photographed under a microscope (Olympus, Tokyo, Japan) at ×200 magnification and the number of cells was counted by the ImageJ software. Each assay was repeated three times.

### RNA-pulldown assay

Biotin-labeled tRF-Val and antisense probes were designed and synthesized by BioSune (Shanghai, China). The RNA-pulldown assay was performed using the RNA-Protein Pull Down Kit (Thermo) according to the manufacturer’s protocol. The proteins were identified with mass spectrometry by the Advanced Medical Research Institute of Shandong University.

### RNA immunoprecipitation (RIP)

RIP assay was performed using the RNA Immunoprecipitation Kit (Geneseed, Guangzhou, China). Briefly, anti-EEF1A1 (Cell Signaling Technology, MA, USA) or anti-IgG (CST) antibody was captured with magnetic beads and incubated with total RNA lysate of AGS cells. Finally, the protein-binding RNA was extracted and purified, and detected by qRT-PCR.

### Western blot (WB) assay

Total proteins of cells were extracted using RIPA lysate (Solarbio, Beijing, China). The cytoplasm and nucleus proteins of cells were extracted using the Subcellular structure Nucleus and Cytoplasm Protein Extraction Kit (Boster, Wuhan, China). The BCA kit (Beyotime, Shanghai, China) was used for protein concentration detection. Briefly, 20 μg loading protein was separated with 10% or 12.5% SDS-PAGE (Epizyme, Shanghai, China), and then transferred onto PVDF membranes (Millipore, MA, USA). The membranes were blocked with Blocking Buffer (Beyotime) for 20 min and incubated at 4 °C overnight with primary antibody ant-EEF1A1 (CST), anti-MDM2 (Santa Cruz Biotechnology, TX, USA), anti-p-MDM2 (Ser166) (abcam, MA, USA), anti-p53 (Proteintech, Wuhan, China), anti-p21 (Proteintech), anti-Bax (CST), anti-Bcl2 (CST). GAPDH (CST) was used as the control for cytoplasmic proteins, and Histone H3 (CST) was used as the control for nuclear proteins. Then the membranes were incubated with the corresponding species secondary antibody (Proteintech) for 2 h. Finally, the blots were visualized by Amersham Imager 600 system (GE, Boston, MA, USA). The relative quantitative values of the bands were measured by ImageJ software. Each assay was repeated three times.

### Immunofluorescence (IF) assay

Briefly, the transfected cells (8 × 10^4^) were suspended in 2 ml medium and seeded in the six-well plate with glass coverslips. And then the cells on the coverslips were washed with PBS, fixed with paraformaldehyde, and permeabilized with 0.2% Triton reagent. The cells were blocked with Bovine Serum Albumin (BSA) (Solarbio) and incubated at 4 °C overnight with primary antibodies ant-EEF1A1 and anti-MDM2. Then the cells were incubated with fluorescent secondary antibody (Proteintech) for 1 h. Nuclei were stained with DAPI reagent. Finally, the cells were photographed by fluorescence microscope (Olympus, Tokyo, Japan).

### Fluorescence in situ hybridization (FISH)

The FISH assay was conducted to detect the subcellular localization of tRF-Val in AGS and MKN-45 cells. Briefly, Cy3 labeled tRF-Val probe was designed and synthesized (GenePharma, Shanghai, China) and hybridized overnight with the cells to be tested based on the manufacturer’s instructions. Nuclei were stained with DAPI reagent. Finally, the cells were photographed by fluorescence microscope (Olympus, Tokyo, Japan).

### Immunoprecipitation (IP) assay

Anti-EEF1A1 and anti-p53 antibodies were combined with Protein A/G PLUS-Agarose beads (Santa Cruz Biotechnology, TX, USA), and then incubated with protein lysate of AGS and MKN-45 cells. The proteins were detected by WB assay with anti-EEF1A1, anti-MDM2, anti-p53, and anti-ubiquitin antibodies.

### Immunohistochemistry (IHC) staining

The IHC staining assay was performed using the IHC Kit (Zsgb Bio, Beijing, China). Briefly, the tissues were fixed with paraformaldehyde, dehydrated, and sectioned. Then the sections were blocked with serum and incubated with primary and secondary antibodies. Finally, the sections were stained with DAB reagent and hematoxylin, and then sealed for observation with the microscope.

### Tumorigenesis assay in vivo

The 4-week-old BALB/c Nude mice were chosen for tumorigenesis assay to study the effect of tRF-Val on tumor growth in vivo. The nude mice were purchased from Charles River Laboratory (Beijing, China) and maintained in the Experimental Animal Center of Shandong Provincial Hospital. The mice were randomly assigned to each group (*n* = 5 mice per group). AGS and MKN-45 cells stably transfected with sh-Val or control sh-NC (4 × 10^6^, 150 μl) were subcutaneously injected into the right upper back of the nude mice. The volumes of the tumors were measured every week. After 4 weeks, the tumors were dissected for weight detection, WB and IHC staining. All animal experiments were approved by the Committee for Ethics of Animal Experiments of Shandong Provincial Hospital.

### Statistical analysis

All statistical analyses were carried out using SPSS 26.0 (IBM, Chicago, USA). Student’s *t*-test or the Mann–Whitney *U* test was performed to analyze the statistical significance between the two groups. Paired *t*-test was performed to compare the expression of tRF-Val in 65 tumor tissues and paired normal tissues. Chi-square test was performed to analyze the correlation between the tRF-Val expressions and clinicopathological variables of the patients. Results were presented as mean ± Standard Deviation. For all analyses, a *P*-value <0.05 was considered statistically significant (*P < 0.05, **P < 0.01, and ***P < 0.001).

## Results

### Types of tRFs and tiRNAs are differentially expressed in four GC tissues and paired normal tissues

**Fig. 1:**
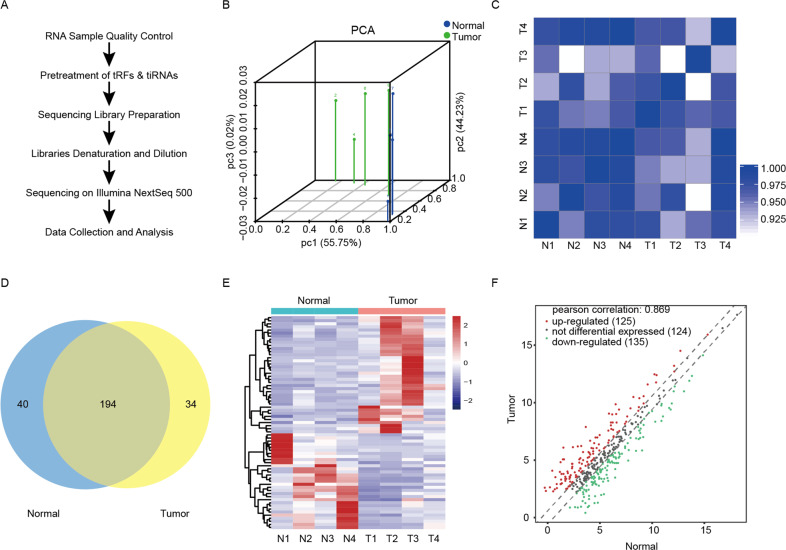
The overview of the tRFs and tiRNAs expressions in four GC tissues and paired normal tissues is shown. **A** The workflow of tRFs and tiRNAs sequencing and data analysis. **B** The principal component analysis (PCA) of tRFs and tiRNAs expressions in tumor and normal samples. **C** The correlation coefficient analysis of tRFs and tiRNAs expressions in tumor and normal samples. **D** Venn diagram of tRFs and tiRNAs expressions in tumor and normal groups. **E** Heatmap of tRFs and tiRNAs in tumor and normal groups. **F** Scatter plot of tRFs and tiRNAs in tumor and normal groups.

To explore whether tRFs and tiRNAs play a key role in the occurrence and progression of GC, four pairs of GC tissues and paired normal tissues were collected for tRFs and tiRNAs-seq experiment. The workflow was summarized in Fig. 1A. The correlation coefficient analysis and principal component analysis (PCA) indicated that there was an ideal correlation between the tumor group and the normal group, and an ideal significantly differential expression between the two groups (Fig. 1B, C). A total of 234 commonly expressed (count-per-million ≥ 20) tRFs and tiRNAs were detected in the normal group and 228 in the tumor group, 194 of which were detected in both the two groups (Fig. 1D). The heatmap and scatter plot showed the expression and distribution of tRFs and tiRNAs in tumor and normal groups, respectively (Fig. 1E, F).

### The expression of tRF-Val, a tRF-3a type, is significantly upregulated in GC

**Fig. 2:**
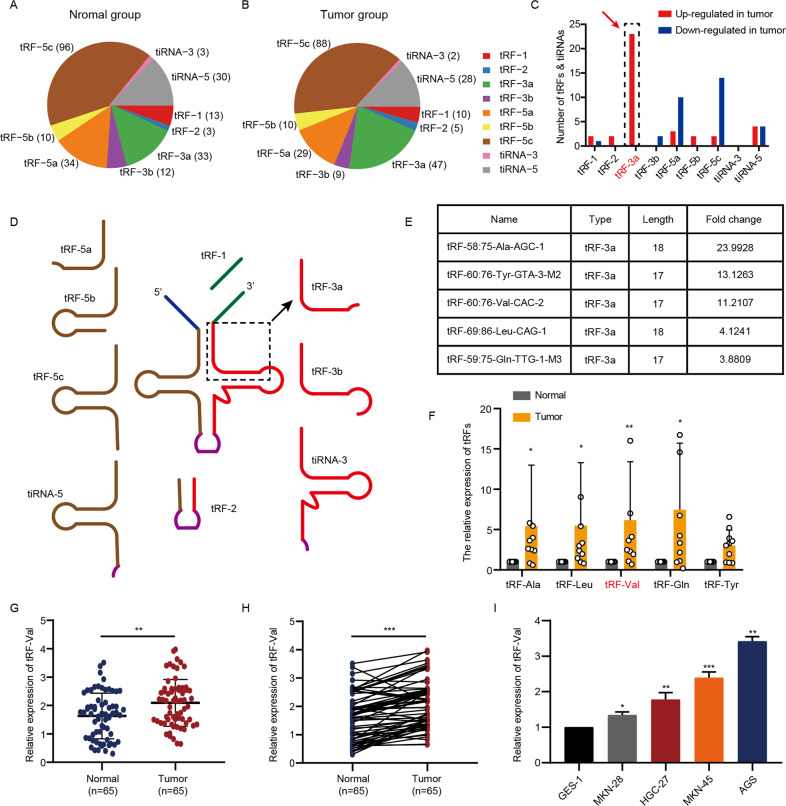
tRF-Val is significantly upregulated in GC. **A**, **B** Distributions of different types of tRFs and tiRNAs in normal and tumor groups. **C** The numbers of significantly upregulated and downregulated (set *P* < 0.05) tRFs and tiRNAs were separately calculated in GC, and tRF-3a was the main upregulated type (23/38). **D** Location of tRF-3a in tRNA-derived fragments. **E** The five of tRF-3a with the highest fold change was selected for further qRT-PCR verification. **F** tRF-Val was the most significantly upregulated tRF in 10 GC tissues compared with matched normal tissues. **G**, **H** Relative expression of tRF-Val in 65 GC tissues and paired normal tissues was detected by qRT-PCR. **I** Relative expression of tRF-Val in GC cell lines and GES-1 was detected by qRT-PCR. Data were shown as mean ± SD. (Mann–Whitney *U* test, student’s *t*-test, and paired *t*-test, *P < 0.05, **P < 0.01, and ***P < 0.001).

To further identify the potential target molecules, the distributions of different types of tRFs and tiRNAs in normal and tumor groups were analyzed (Fig. 2A, B). The result indicated that tRF-3a, tRF-5a, tRF-5c, and tiRNA-5 were the main types detected in GC and normal tissues. Moreover, the numbers of significantly upregulated and downregulated (set *P* < 0.05) tRFs and tiRNAs were separately calculated in GC, and tRF-3a was the main upregulated type (23/38), while tRF-5a (10/31) and tRF-5c (14/31) were the main downregulated types in GC (Fig. 2C), indicating that tRF-3a may play a more important role in the occurrence and progression of GC. Therefore, tRF-3a was chosen as the potential carcinogenic tRFs (Fig. 2D), and five of them with the highest fold change (excluding highly repetitive sequences) were selected for further qRT-PCR verification in a small number of tissue samples (Fig. 2E). The results showed that tRF-58:75-Ala-AGC-1, tRF-69:86-Leu-CAG-1, tRF-60:76-Val-CAC-2, and tRF-59:75-Gln-TTG-1-M3 were significantly upregulated and tRF-60:76-Val-CAC-2 (hereinafter referred to as tRF-Val) was the most significantly upregulated one (*P* = 0.002) in 10 GC tissues compared with matched normal tissues (Fig. 2F). In addition, the expression of tRF-Val was detected in 65 GC tissues and matched normal tissues with qRT-PCR, and the result showed that tRF-Val expression was significantly higher in GC tissues than in normal tissues (Fig. 2G, H). Correlation analyses revealed that tRF-Val expression was positively correlated with tumor size and the depth of tumor invasion in GC tissues (Table 1). Furthermore, the expression of tRF-Val was detected in GC cell lines MKN-28, MKN-45, HGC-27, and AGS, related to GES-1 cell line. The result showed that the expressions of tRF-Val in GC cell lines were all significantly higher than those in GES-1 cell line (Fig. 2I). Among GC cell lines, AGS and MKN-45 were the two cell lines with the highest expression of tRF-Val, so we selected the two cell lines for the biological function study and downstream mechanism research.

**Table 1:** Correlation between tRF-Val expression and clinicopathological characteristics in 65 GC patients.

Parameters	Cases	tRF-Val expression		*P*-value
		Low	High	
Total	65	32	33	
Age				0.540
<60	22	12	10	
≥60	43	20	23	
Gender				0.170
Male	40	17	23	
Female	25	15	10	
Tumor invasion				0.040*
T1–T2	17	12	5	
T3–T4	48	20	28	
Lymph node metastasis				0.255
N0	22	13	9	
N1–N3	43	19	24	
TNM stage				0.174
I–II	29	17	12	
III	36	15	21	
Tumor size				0.004*
<5 cm	31	21	10	
≥5 cm	34	11	23	

*Statistically significant.

### tRF-Val promotes proliferation and invasion and inhibits apoptosis in GC cells

**Fig. 3:**
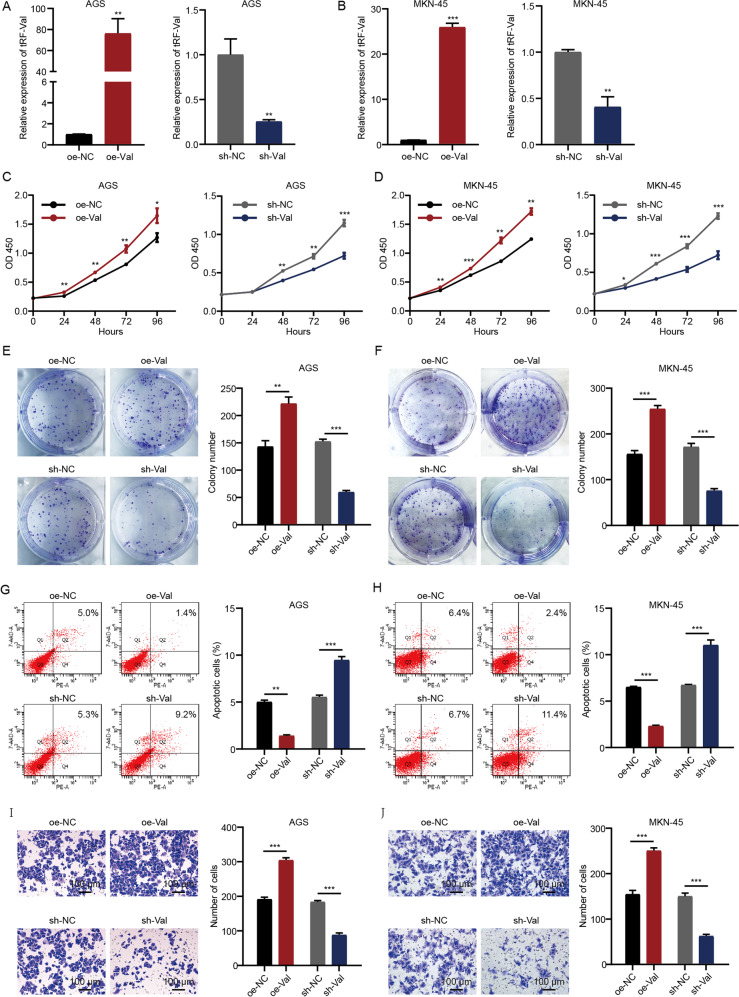
tRF-Val promotes proliferation and invasion and inhibited apoptosis of GC cells in vitro. **A**, **B** The knockdown and overexpression efficiencies of tRF-Val in AGS and MKN-45 cells were verified by qRT-PCR. **C**, **D** CCK-8 assay was performed to detect the proliferation of oe-NC, oe-Val, sh-NC, and sh-Val transfected AGS and MKN-45 cells. **E**, **F** Colony formation assay was performed to detect the proliferation of oe-NC, oe-Val, sh-NC, and sh-Val transfected AGS and MKN-45 cells. **G**, **H** Flow cytometry analysis was performed to detect the apoptosis of oe-NC, oe-Val, sh-NC, and sh-Val transfected AGS and MKN-45 cells. **I**, **J** Transwell assay was performed to detect the invasion of oe-NC, oe-Val, sh-NC, and sh-Val transfected AGS and MKN-45 cells. The scale bar, 100 μm. Data were shown as mean ± SD. (Student’s *t*-test, *P < 0.05, **P < 0.01, and ***P < 0.001). NC means negative control, sh means short hairpin RNA, Val means tRF-Val, and oe means overexpression.

To further explore the biological effects of tRF-Val in GC cells, the tRF-Val overexpression and knockdown models were constructed in AGS and MKN-45 cells. The knockdown and overexpression efficiencies of the two cells were verified by qRT-PCR assay (Fig. 3A, B). CCK-8 assay results indicated that tRF-Val overexpression significantly promoted the proliferation of AGS and MKN-45 cells. In contrast, tRF-Val knockdown significantly inhibited the proliferation of AGS and MKN-45 cells (Fig. 3C, D). Colony formation assay results indicated that tRF-Val overexpression significantly promoted colony formation in AGS and MKN-45 cells, while tRF-Val knockdown significantly inhibited colony formation in AGS and MKN-45 cells (Fig. 3E, F). In addition, tRF-Val overexpression reduced the apoptosis rate of AGS and MKN-45 cells, while tRF-Val knockdown increased the apoptosis rate of AGS and MKN-45 cells (Fig. 3G, H). Transwell invasion assay results indicated that tRF-Val overexpression significantly promoted the invasion of AGS and MKN-45 cells, whereas tRF-Val knockdown significantly inhibited the invasion of AGS and MKN-45 cells (Fig. 3I, J). To summarize, tRF-Val promoted proliferation and invasion and inhibited apoptosis in GC cells in vitro.

### tRF-Val directly binds to EEF1A1 protein and promotes its transport into the nucleus

**Fig. 4:**
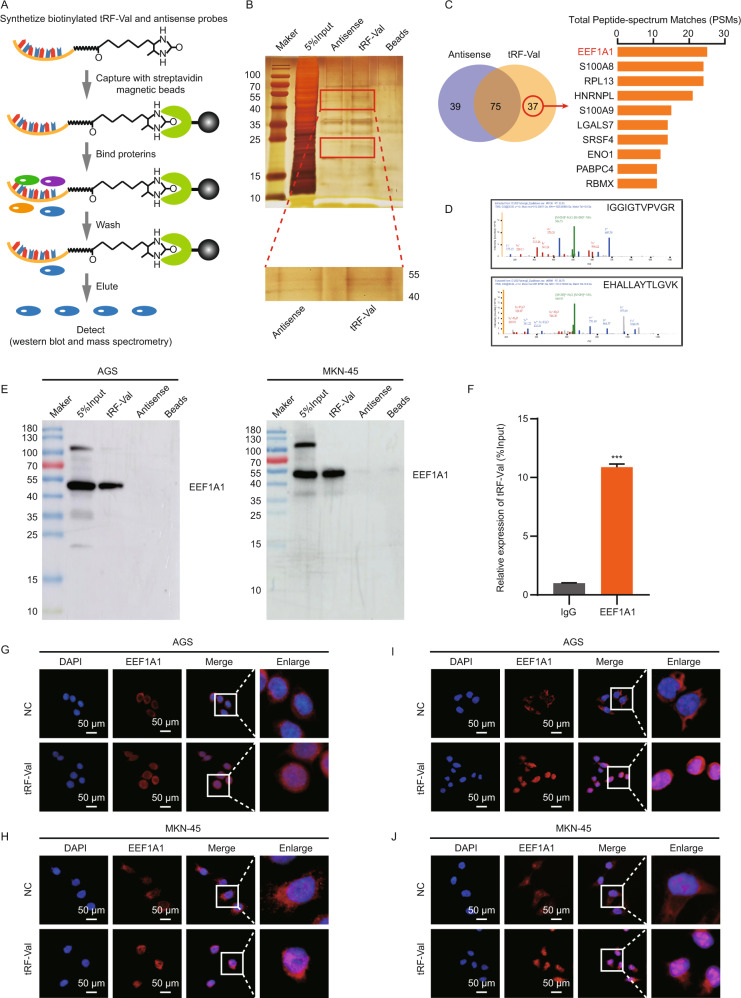
tRF-Val directly binds to EEF1A1 protein and promotes its transport into nucleus. **A** The workflow of tRF-Val pulldown assay. **B** Silver staining assay was performed to detect the differential proteins obtained by the pulldown assay in AGS cells. **C** The top 10 proteins with the highest PSMs scoring identified by mass spectrometry were shown, and EEF1A1 was identified as a tRF-Val binding protein. **D** The two specific peptides of EEF1A1 identified by mass spectrometry. **E** Independent RNA-pulldown assays and WB assays confirmed the interaction of tRF-Val and EEF1A1 in AGS and MKN-45 cells, respectively. **F** RIP assay was performed to detect the interaction of tRF-Val and EEF1A1 in AGS cells. **G**, **H** IF assay proved that tRF-Val significantly enhanced the fluorescence of EEF1A1 in the nucleus of AGS and MKN-45 cells. The scale bar, 50 μm. **I**, **J** FISH assay confirmed that tRF-Val overexpression mainly enhanced its fluorescence in the nucleus. The scale bar, 50 μm. Data were shown as mean ± SD. (Student’s *t*-test, ***P < 0.001). PSMs means peptide-spectrum matches.

It has been widely reported that tRFs participated in tumor progression by directly binding target RNAs or proteins [15], so we speculated whether tRF-Val could play a carcinogenic role through binding target proteins. To verify the hypothesis, RNA-pulldown assay was performed (Fig. 4A), and the AGS cell line with the highest expression of tRF-Val was selected as the target cell line. The biotinylated tRF-Val and the antisense probes were incubated with the protein lysate of AGS cells, and differential protein bands were identified by silver staining assay. The result indicated that there were two distinct bands in the 40–55 KD and 15–25 KD positions on the silver staining gel (Fig. 4B). Furthermore, 37 specific binding proteins were obtained by mass spectrometry in the tRF-Val band, but they did not exist in the antisense sequence (Supplementary Table 3). Among them, EEF1A1 protein with a molecular weight of 50 KD was the highest-scoring tRF-Val binding protein (Fig. 4C, D). In addition, independent RNA-pulldown assays and WB assays also confirmed the interaction of tRF-Val and EEF1A1 in AGS and MKN-45 cells, respectively (Fig. 4E). RIP assay results also proved that tRF-Val could be enriched by EEF1A1 antibody group, compared with IgG antibody group in AGS cells (Fig. 4F).

The above studies have proved that tRF-Val could specifically bind to EEF1A1, but the effects need to be further explored. The qRT-PCR assay indicated that tRF-Val overexpression and knockdown had no significant effect on the EEF1A1 mRNA expression in AGS and MKN-45 cells (Supplementary Fig. 1A). Subsequently, the EEF1A1 overexpression and knockdown models were constructed in AGS and MKN-45 cells, and the qRT-PCR assay indicated that EEF1A1 overexpression and knockdown also had no significant effect on the tRF-Val expression (Supplementary Fig. 1B, C). Moreover, the WB assay indicated that tRF-Val overexpression and knockdown did not significantly affect the total EEF1A1 protein expression, but may promote its transport from cytoplasm to nucleus (Supplementary Fig. 1D). The IF assay also proved that tRF-Val significantly enhanced the fluorescence of EEF1A1 in the nucleus of AGS and MKN-45 cells (Fig. 4G, H). Interestingly, the FISH assay confirmed that tRF-Val overexpression also mainly enhanced its fluorescence in the nucleus (Fig. 4I, J), which was the same trend as EEF1A1. Furthermore, the colony formation rescue assay indicated that the growth-promoting effect of tRF-Val overexpression on GC cells AGS and MKN-45 could be reversed by the knockdown of EEF1A1 (Supplementary Fig. 1E). Through the above experiments, we concluded that tRF-Val may promote the progression of GC by mediating EEF1A1 transport to the nucleus.

### EEF1A1 promotes growth and inhibits apoptosis in GC cells

**Fig. 5:**
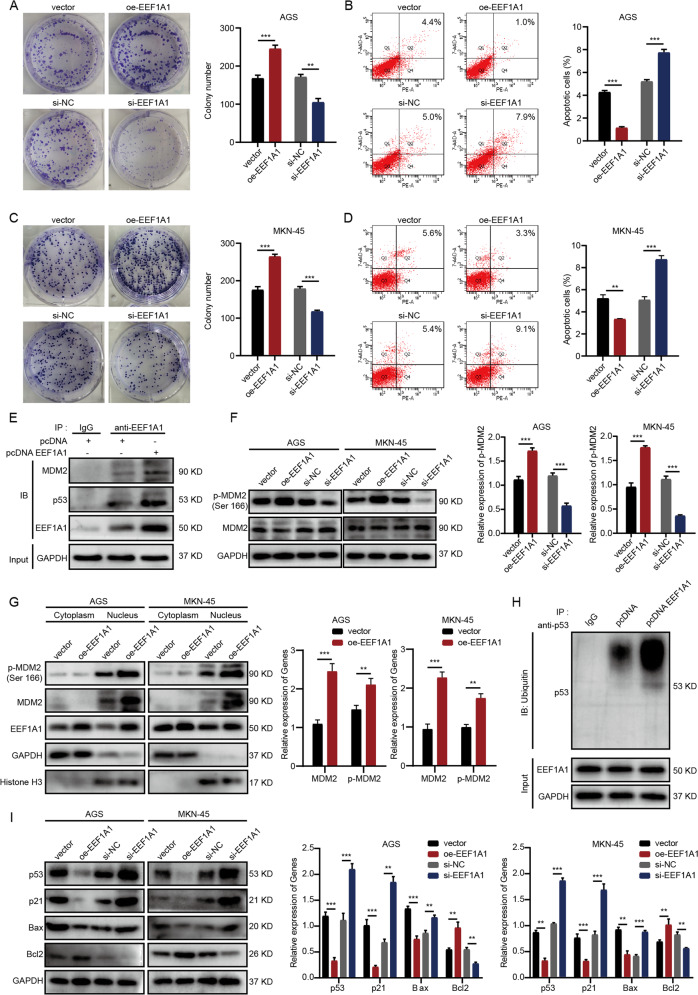
EEF1A1 combines with MDM2-p53 complex and enhances the downstream effects of MDM2. **A**, **C** Colony formation assay was performed to detect the proliferation of vector, oe-EEF1A1, si-NC, and si-EEF1A1 transfected AGS and MKN-45 cells. **B**, **D** Flow cytometry analysis was performed to detect the apoptosis of vector, oe-EEF1A1, si-NC, and si-EEF1A1 transfected AGS and MKN-45 cells. **E** Co-IP assay was performed to detect the combination of EEF1A1 and MDM2-p53 complex in AGS cells, and more MDM2-p53 was co-immunoprecipitated by anti-EEF1A1 antibody in the pcDNA EEF1A1 lane compared with pcDNA lane. **F** The p-MDM2 was detected in vector, oe-EEF1A1, si-NC, and si-EEF1A1 transfected AGS and MKN-45 cells by WB. **G** WB assay proved that EEF1A1 overexpression significantly promoted the nuclear localization of MDM2 and p-MDM2. **H** EEF1A1 overexpression significantly promoted the ubiquitination of p53. **I** WB was performed to detect the expressions of MDM2 downstream molecules (p53, p21, Bax, and Bcl2) in AGS and MKN-45 cells. Data were shown as mean ± SD. (Student’s *t*-test, **P < 0.01 and ***P < 0.001). si means small interfering RNA.

It had been confirmed in previous studies that EEF1A1 made oncogenic functions in various tumors, such as lung cancer [20], renal cell carcinoma [21], hepatocellular carcinoma [22], and GC [23, 24], etc. Besides, EEF1A1 expression level is associated with poor prognosis in GC based on the Cancer Genome Atlas (TCGA) database (Supplementary Fig. 1F). To verify the carcinogenic effect of EEF1A1 in GC, the biological function experiments in AGS and MKN-45 cells were conducted. Colony formation assay results indicated that EEF1A1 overexpression significantly promoted colony formation, while its knockdown significantly inhibited colony formation in AGS and MKN-45 cells (Fig. 5A, C). In addition, EEF1A1 overexpression significantly inhibited apoptosis, while EEF1A1 knockdown promoted apoptosis in AGS and MKN-45 cells (Fig. 5B, D). This suggested that EEF1A1 could promote the progression of GC, which was consistent with the previous studies [24].

### EEF1A1 interacts with MDM2-p53 complex and enhances the effects of MDM2

The above studies have confirmed that EEF1A1 could promote the growth and inhibit the apoptosis of GC cells, but the specific mechanism was still not clear. It was reported that EEF1A1 enhanced the function of interacting proteins by acting as molecular chaperones [23–29] (summarized in the Supplementary Fig. 2), and MDM2 was the most commonly reported interacting protein of EEF1A1 which had been studied in WI38, H1299, Cortical neuron, and HeLa cells, etc [25–27]. As a nuclear-localized E3 ubiquitin ligase, MDM2 can promote tumor formation by targeting tumor suppressor protein p53 and mediating its ubiquitination [30, 31]. It was reported that the combination of EEF1A1 and MDM2-p53 complex promoted the phosphorylation of MDMD2 at the Ser166 residue [26, 27], which was conducive to maintain the nuclear localization of MDM2 and enhanced its ubiquitin ligase activity, leading to the degradation of p53 [32–34]. Based on the above conclusions, we speculated whether EEF1A1 caused tumor progression in GC also by interacting with MDM2 and enhancing its effects. To verify the hypothesis, Co-IP assay was performed (Fig. 5E, Supplementary Fig. 3). The results showed that anti-EEF1A1 antibody co-immunoprecipitated MDM2-p53 from pcDNA EEF1A1 and control pcDNA plasmids transfected AGS and MKN-45 cell extracts, and meaningfully, more MDM2-p53 was co-immunoprecipitated in the pcDNA EEF1A1 lane. MDM2-p53 was not co-immunoprecipitated by IgG antibody as expected. This indicated that EEF1A1 could combine with MDM2-p53 complex in GC cells. Besides, EEF1A1 overexpression significantly promoted the phosphorylation of MDM2 at the Ser166 residue in AGS and MKN-45 cells; on the contrary, EEF1A1 knockdown significantly reduced the phosphorylation of MDM2 (Fig. 5F). Moreover, the levels of MDM2 and p-MDM2 protein in the nucleus were significantly upregulated when EEF1A1 was overexpressed in AGS and MKN-45 cells (Fig. 5G), which was also consistent with our assumption. To verify the reliability, we further studied the effects of EEF1A1 on the downstream targets of MDM2. The results showed that EEF1A1 overexpression significantly promoted the ubiquitination of p53 (Fig. 5H). Furthermore, the WB assays showed that EEF1A1 overexpression significantly decreased the expression of p53, p21, and Bax, and increased the expression of Bcl2 in AGS and MKN-45 cells, while the knockdown of EEF1A1 showed just the opposite result (Fig. 5I). To sum up, EEF1A1 could combine with MDM2-p53 complex and enhance the downstream effects of MDM2, leading to GC progression.

### tRF-Val regulates the MDM2/p53 pathway by promoting the interaction between EEF1A1 and MDM2

**Fig. 6:**
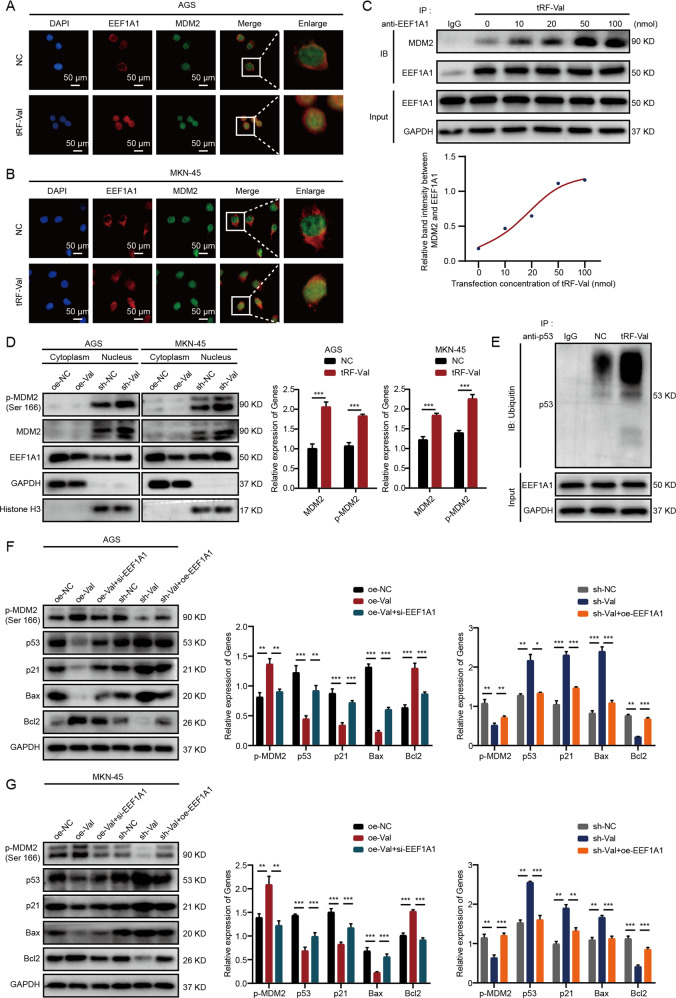
tRF-Val regulates the MDM2/p53 pathway by promoting the interaction between EEF1A1 and MDM2. **A**, **B** IF assay showed that tRF-Val promoted the colocalization of EEF1A1 and MDM2 in the nucleus of AGS and MKN-45 cells. The scale bar, 50 μm. **C** Co-IP assay showed that the amount of MDM2 co-immunoprecipitated by anti-EEF1A1 antibody increased in a gradient with the increase of tRF-Val transfection concentration in AGS cells. **D** WB assay proved that tRF-Val overexpression significantly promoted the nuclear localization of MDM2 and p-MDM2. **E** tRF-Val overexpression significantly promoted the ubiquitination of p53. **F**, **G** WB rescue experiments showed that the regulatory effect of tRF-Val on p-MDM2 and its downstream target molecules could be reversed by the knockdown and overexpression of EEF1A1 in AGS and MKN-45 cells. Data were shown as mean ± SD. (Student’s *t*-test, *P < 0.05, **P < 0.01, and ***P < 0.001).

The above experiments result have confirmed that tRF-Val mediated the transport of EEF1A1 into the nucleus, and EEF1A1 could interact with the nuclear-localized MDM2 and mediate its downstream effects. Therefore, we speculated that tRF-Val could regulate the MDM2/p53 pathway by promoting the interaction between EEF1A1 and MDM2, which eventually led to the progression of GC. To verify the hypothesis, IF and Co-IP assays were performed. The results of IF assay showed that tRF-Val promoted the colocalization of EEF1A1 and MDM2 in the nucleus of AGS and MKN-45 cells (Fig. 6A, B). The results of Co-IP assay showed that after transfection with tRF-Val at the concentration of 0, 10, 20, 50, and 100 nmol in AGS cells, the amount of MDM2 co-immunoprecipitated by anti-EEF1A1 antibody increased in a gradient (Fig. 6C). More importantly, tRF-Val also significantly increased the levels of MDM2 and p-mdm2 protein in the nucleus of AGS and MKN-45 cells (Fig. 6D), and significantly promoted the ubiquitination of p53 (Fig. 6E). Furthermore, the WB rescue experiments showed that tRF-Val overexpression significantly increased the expression of p-MDM2 and Bcl2, and decreased the expression of p53, p21, and Bax in AGS and MKN-45 cells, while the knockdown of tRF-Val showed just the opposite result. Simultaneously, the effects of the knockdown or the overexpression of tRF-Val on MDM2/P53 pathway were reversed by EEF1A1 overexpression and silence respectively (Fig. 6F, G). Besides, CCK-8 rescue assays and colony formation rescue assays indicated that the growth-promoting effect of tRF-Val overexpression on GC cells AGS and MKN-45 could be reversed by the knockdown of MDM2 (Supplementary Fig. 4A–E). Collectively, tRF-Val regulated the MDM2/p53 pathway by promoting the interaction between EEF1A1 and MDM2.

### Knockdown of tRF-Val inhibits the growth of GC in vivo

**Fig. 7:**
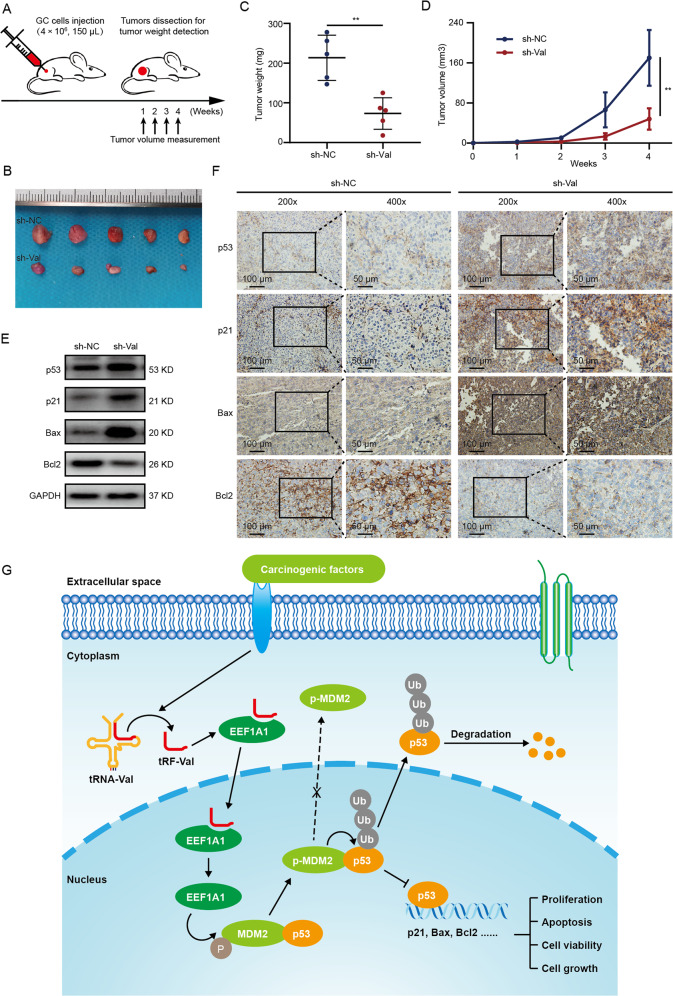
Knockdown of tRF-Val inhibits the growth of GC in vivo. **A** The workflow of subcutaneous tumorigenesis. **B** The tumors of nude mice were dissected at 4 weeks after AGS cells injection in sh-NC and sh-Val groups (*n* = 5 mice per group). **C**, **D** The weights and volumes of the tumors were significantly decreased in the sh-Val group than those in the sh-NC group. **E**, **F** The WB and IHC results indicated that expressions of p53, p21, and Bax were upregulated, while that expression of Bcl2 was downregulated in the sh-Val group. The scale bar, 100 μm (200×); 50 μm (400×). **G** The schematic diagram of the mechanisms that tRF-Val directly bound to EEF1A1, mediated its entry into the nucleus and promoted its binding with MDM2, thus inhibited the downstream molecular pathway of p53 and promote GC progression. Data were shown as mean ± SD. (Student’s *t*-test, **P < 0.01).

To further explore the effects of tRF-Val in vivo, a subcutaneous tumorigenesis experiment was carried out in nude mice (Fig. 7A). AGS and MKN-45 cells stably transfected with sh-Val or control sh-NC (4 × 10^6^, 150 μl) were subcutaneously injected into the right upper back of the nude mice (*n* = 5 mice per group). The volumes of the tumors were measured every week. After 4 weeks, the tumors were dissected (Fig. 7B, Supplementary Fig. 5A). The weights and volumes of the tumors significantly decreased in the sh-Val group than those in the sh-NC group (Fig. 7C, D; Supplementary Fig. 5B, C). The subcutaneous tumors in the sh-Val and sh-NC groups were further deployed for WB and IHC assays. WB assay results indicated that expressions of p53, p21, and Bax were upregulated, while the expression of Bcl2 was downregulated in the sh-Val group (Fig. 7E). And the results of IHC assay were consistent with those of the WB assay (Fig. 7F). Collectively, these results demonstrated that knockdown of tRF-Val inhibited the growth of GC in vivo.

Taken together, tRF-Val directly bound to EEF1A1, mediated its transport into the nucleus and promoted its interaction with MDM2, thus inhibiting the downstream molecular pathway of p53 and ultimately promoting tumor progression in GC (Fig. 7G).

## Discussion

At present, it is commonly believed that tRFs and tiRNAs were formed by the specific and selective shear of tRNAs under certain pressure stimulation, rather than by random degradation of tRNA [15, 16]. An increasing number of reports show that tRFs contribute to the biological process of a variety of human cancers, such as thyroid cancer [17], breast cancer [18], and bladder cancer [35], etc. However, the evidence for the mechanisms of tRFs and tiRNAs in the occurrence and development of GC is still insufficient, so we performed the tRFs and tiRNAs sequencing in GC.

In this study, the sequencing results showed that tRF-3a was the main upregulated type, while tRF-5a and tRF-5c were the main downregulated types in GC, indicating that tRF-3a may play an important role in the occurrence and progression of GC, while tRF-5a and tRF-5c may be involved in the inhibition of GC. It is the first time to obtain direct evidence of the different effects of different types of tRFs on tumors, and such results may not be accidental. Besides, we identified a high-expressed tRF-3a type fragment, tRF-Val in GC for the first time, and its high expression was positively correlated with advanced clinical features of GC patients. Subsequently, the biological functions and carcinogenic mechanisms of tRF-Val were systematically and comprehensively explored in the present study.

Firstly, the CCK-8 assay, colony formation assay, transwell assay, and flow cytometry assay totally proved that tRF-Val promoted proliferation and invasion, and inhibited apoptosis in GC cells, indicating that tRF-Val was a novel potential oncogene in GC. Secondly, RNA-pulldown assay and RIP assay coordinately confirmed the combination of tRF-Val and EEF1A1. Thirdly, sufficient evidence proved that tRF-Val mediated EEF1A1 transport into the nucleus, thus enhancing the interaction between EEF1A1 and nuclear-localized MDM2-p53 complex, and inhibiting the downstream molecular pathway of p53. Based on the above evidence, the results suggested the importance of tRF-Val as a novel oncogene and therapeutic target in GC.

EEF1A1 as a MDM2-binding partner was first reported by Frum R et al. in WI38 and H1299 lung cancer cells [25]. However, the researchers did not detect HDM2-p53 interaction in the follow-up mechanism experiments due to the deficiency of endogenous p53 in H1299 cells. Tsai N et al. systematically studied the interaction of EEF1A1 and MDM2, and first proved that EEF1A1 enhanced the nuclear localization and function of MDM2 by promoting the phosphorylation of MDM2 at the Ser166 residue [26]. Then another researcher Blanch A also reported EEF1A1 may inhibit p53-dependent apoptosis in Hela and SaOS2 cells by mediating the MDM2-p53 interaction [27]. In this GC research paper, we demonstrated the binding of EEF1A1 with MDM2-p53 complex by Co-IP and IF experiments, and the biological role of EEF1A1 in regulating p53 ubiquitination by promoting MDM2 phosphorylation. Therefore, we speculate that EEF1A1 could promote the MDM2-p53 interaction, which may be a common pathway in the pan-cancer progression. Of course, this speculation deserves more cancer studies.

p53 plays a central role in the cell responses to external stimuli and stress [36]. As the core gene of tumor suppressors, p53 could maintain the normal physiological function of cells by regulating a variety of cellular pathways, such as apoptosis, damage repairing, cell cycle arresting, and so on [37]. The inactivation of tumor suppressor p53 is an important cause of cancers [38–40]. This present study provides a brand-new explanation for p53-mediated gastric carcinogenesis.

However, there are several limitations in the original research. Firstly, the four GC tissues and paired normal tissues used for sequencing were taken from a homogenous population in Shandong Provincial Hospital, and the sequencing results might not cover all upregulated and downregulated tRFs. Secondly, we did not explore whether tRF-Val participated in the regulatory mechanism of mutant oncogenic p53. Although a recent sequencing study indicated the presence of the p53 Arg110Cys mutation in MKN-45 cells, it is listed as an indeterminate mutant in the ACMG (the American College of Medical Genetics and Genomics) guidelines [41], and the guidelines state that this missense mutation may not affect the structure and function of the p53 protein. Moreover, p53 in MKN-45 cells is not listed as a germline mutation in IARC (International Agency for Research on Cancer) TP53 database [42]. Therefore, in most studies, p53 in MKN-45 cells was still classified as a tumor suppressor protein [43–45]. Thirdly, further studies are needed to investigate if tRF-Val could interact with mRNAs and other proteins.

In conclusion, this paper provided sufficient evidence that tRF-Val was significantly upregulated in GC and it could function as an oncogene. Mechanistically, we identified that tRF-Val directly bound to EEF1A1, mediated its transport into the nucleus, and promoted its interaction with MDM2, thus inhibiting the downstream molecular pathway of p53 and promoting GC progression. These findings provided a new potential therapeutic target for GC and a new explanation for the occurrence of GC.

## Supplementary information

Supplementary Figure 1

Supplementary Figure 2

Supplementary Figure 3

Supplementary Figure 4

Supplementary Figure 5

Supplementary Figure Legends

The primer sequences of the genes in this experiment

Supplementary Table 2

The 37 specific tRF-Val binding proteins identified by mass spectrometry are shown.

Original Data File

aj-checklist

cdd-author-contribution-form

## Data Availability

The datasets used and/or analyzed during the current study are available from the corresponding author on reasonable request.
