# Supplementary figures and images for: A novel 3’tRNA-derived fragment tRF-Val promotes proliferation and inhibits apoptosis by targeting EEF1A1 in gastric cancer
Source: Cell Death Dis. 2022 May 18;13(5):471. doi: 10.1038/s41419-022-04930-6 (PMC9117658; doi:10.1038/s41419-022-04930-6)

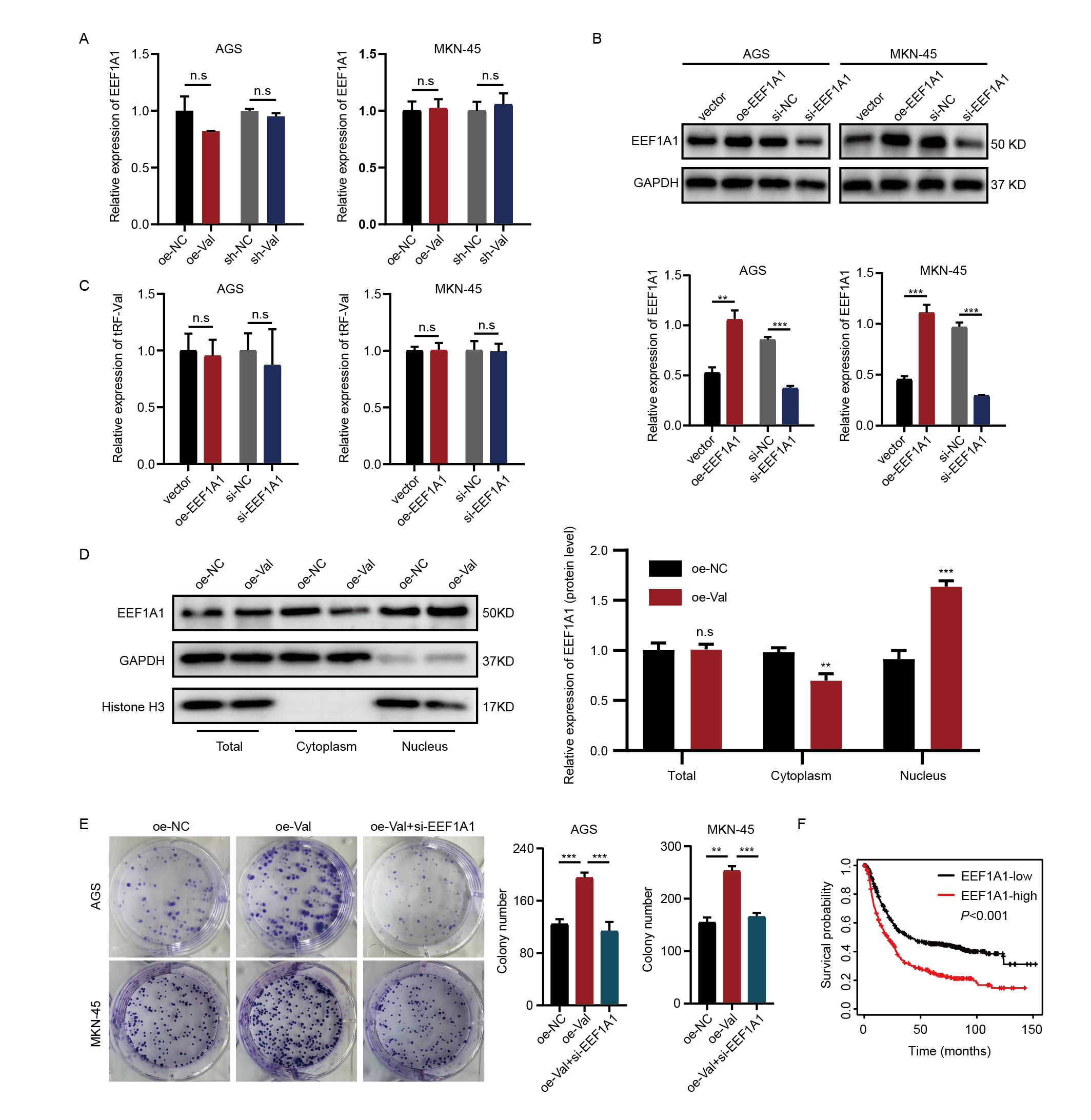

Supplement: Supplementary file 1 — Supplementary Figure 1 [file 41419_2022_4930_MOESM1_ESM.tif]

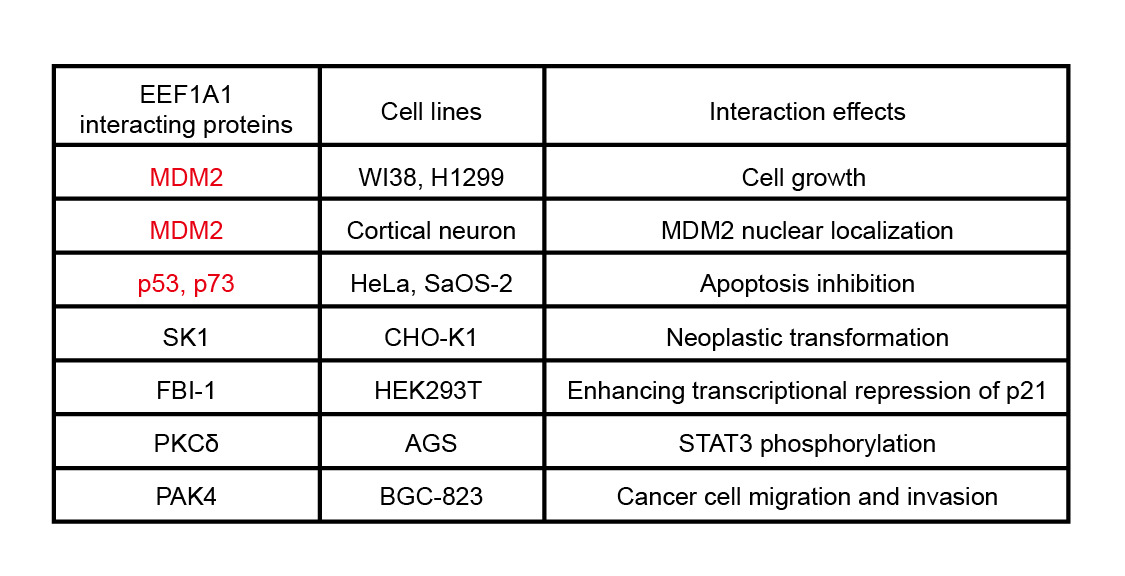

Supplement: Supplementary file 2 — Supplementary Figure 2 [file 41419_2022_4930_MOESM2_ESM.tif]

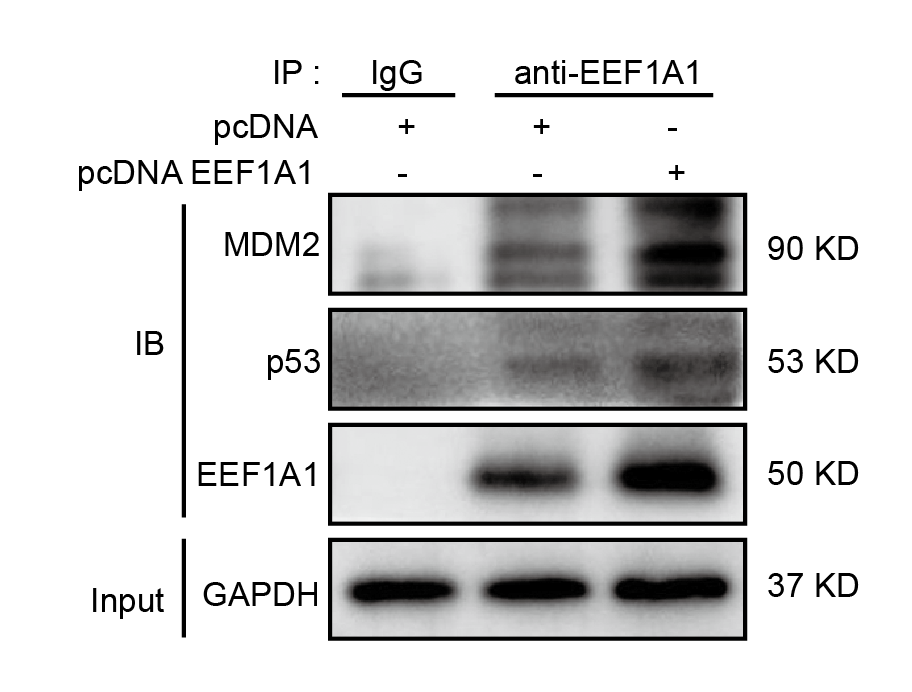

Supplement: Supplementary file 3 — Supplementary Figure 3 [file 41419_2022_4930_MOESM3_ESM.tif]

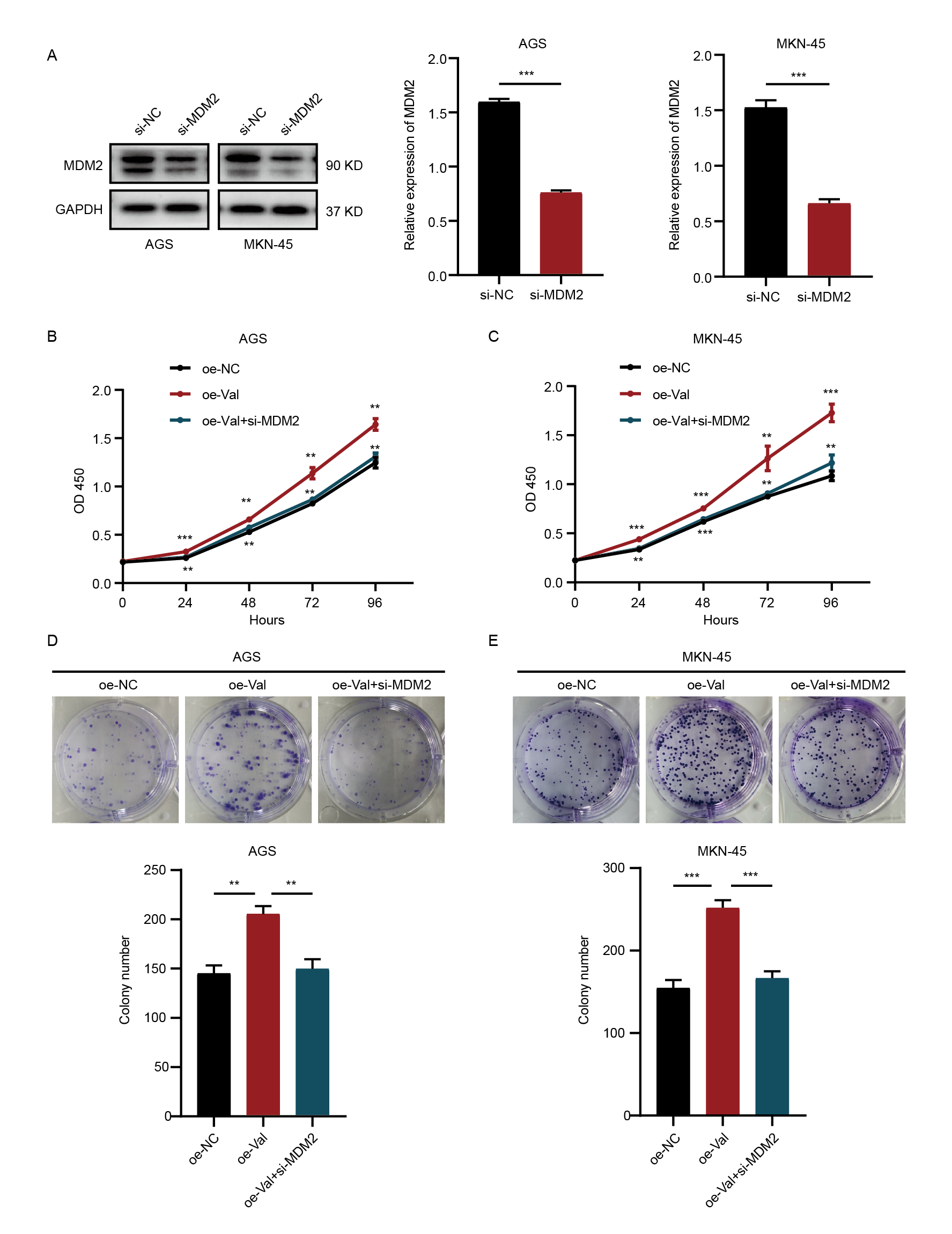

Supplement: Supplementary file 4 — Supplementary Figure 4 [file 41419_2022_4930_MOESM4_ESM.tif]

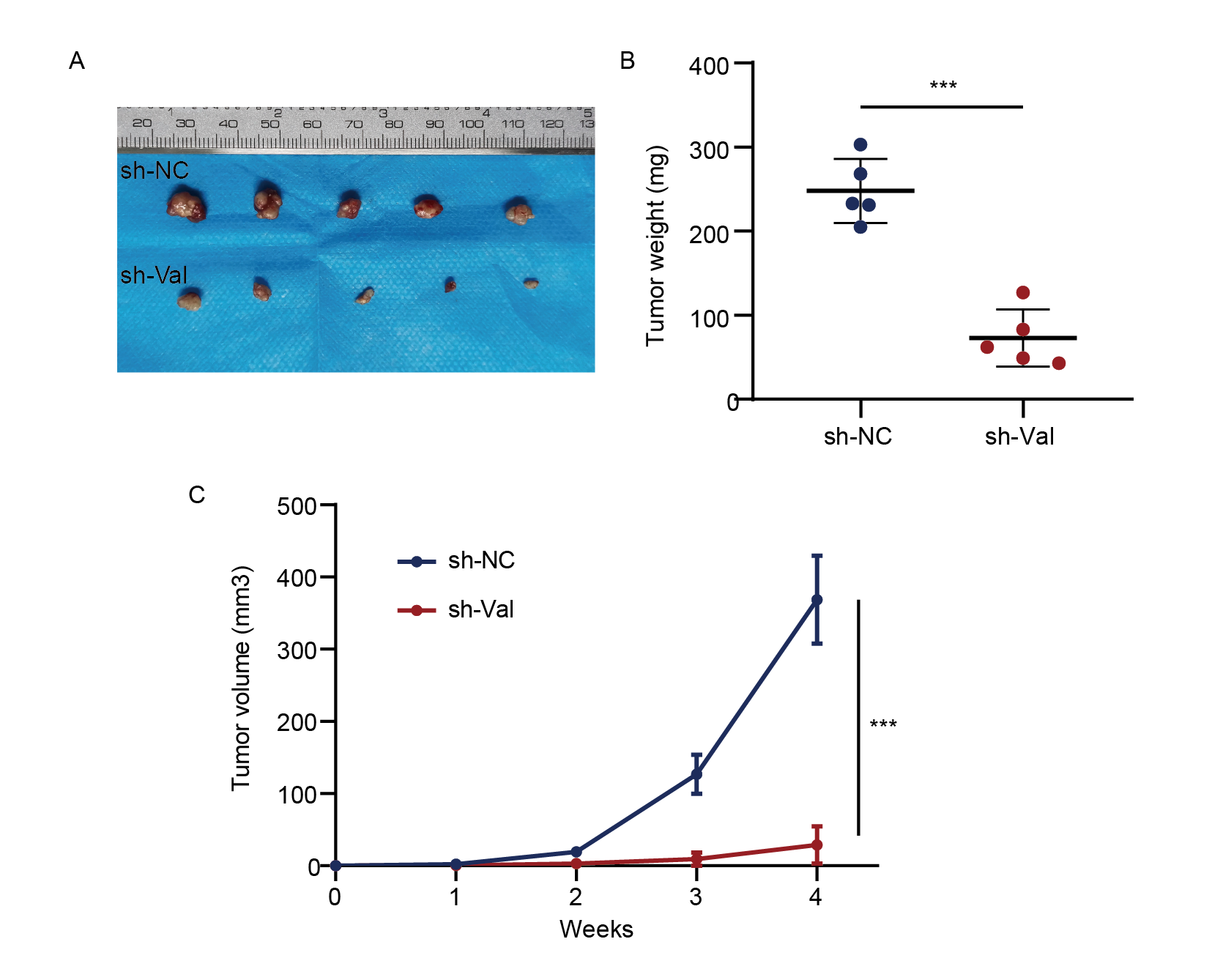

Supplement: Supplementary file 5 — Supplementary Figure 5 [file 41419_2022_4930_MOESM5_ESM.tif]

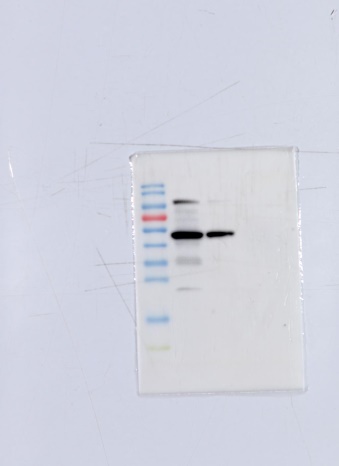

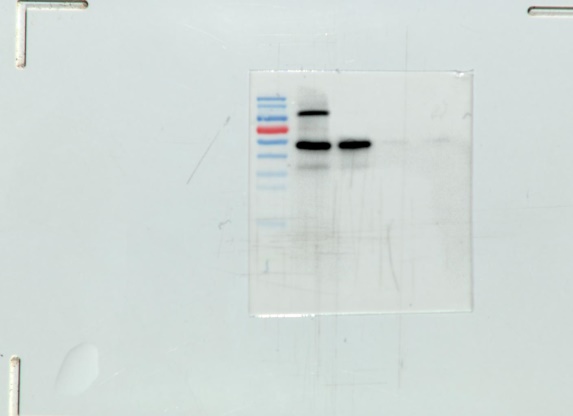


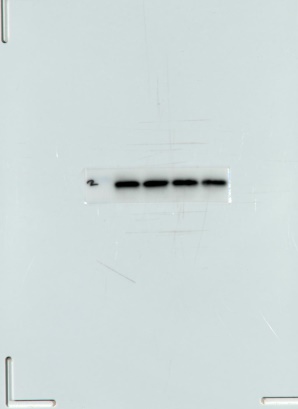

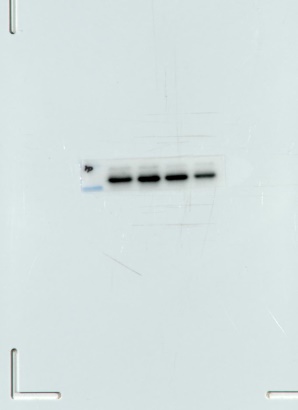

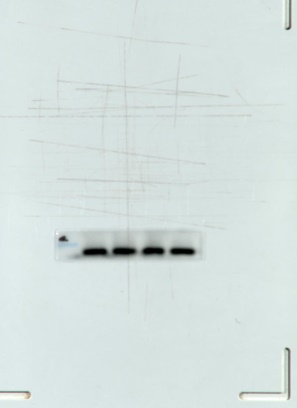

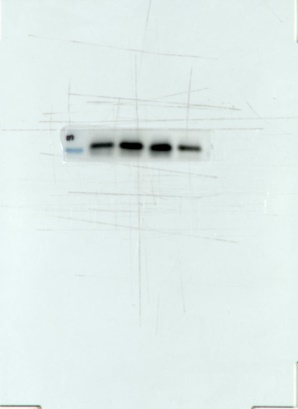


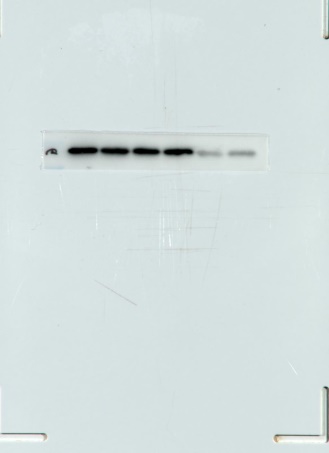

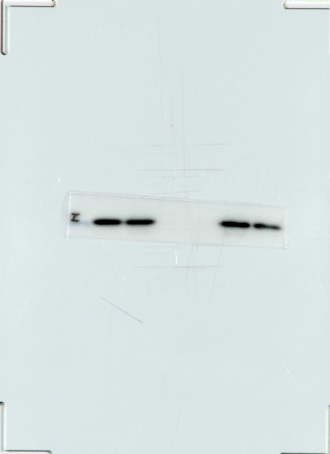

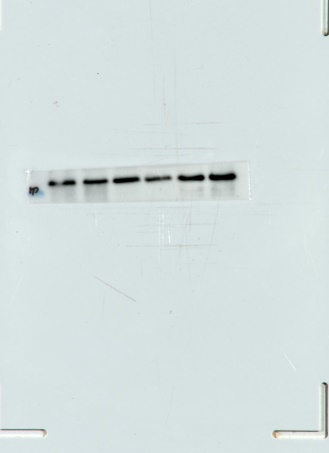


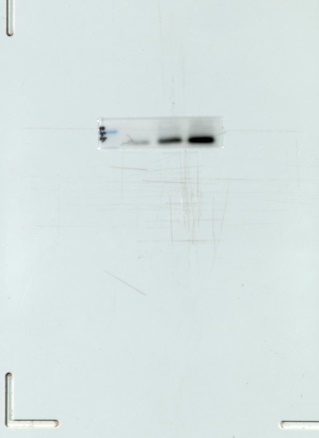

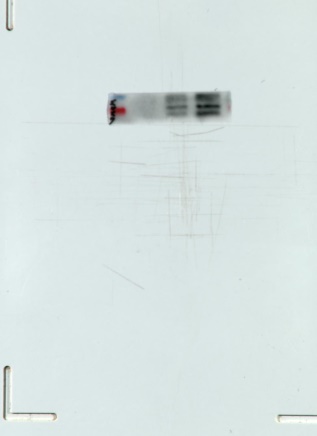

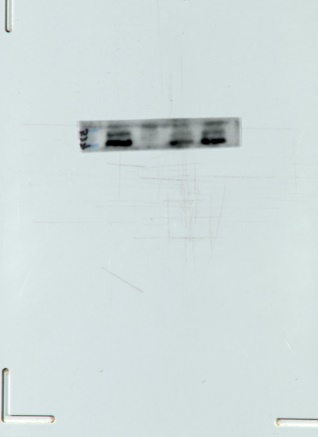

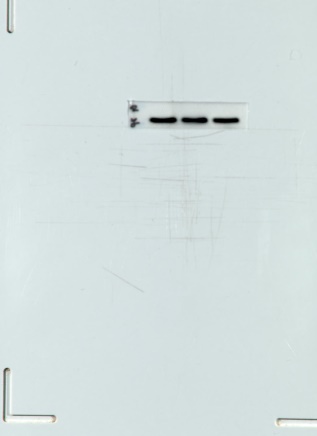


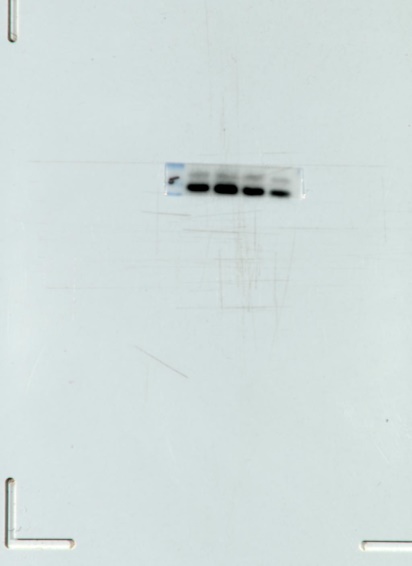

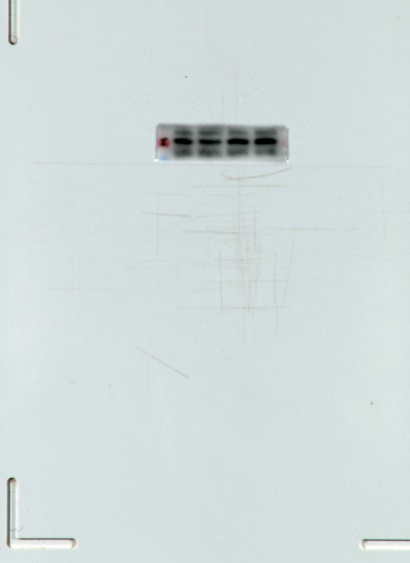

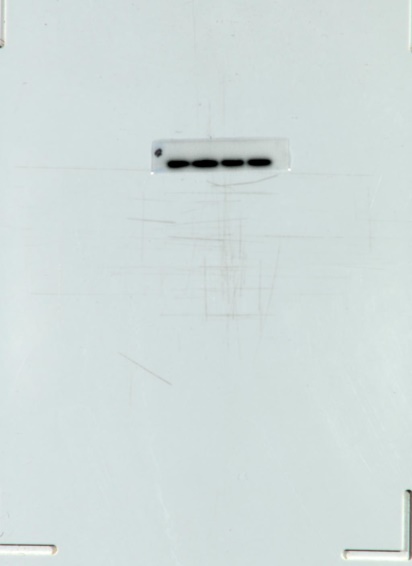


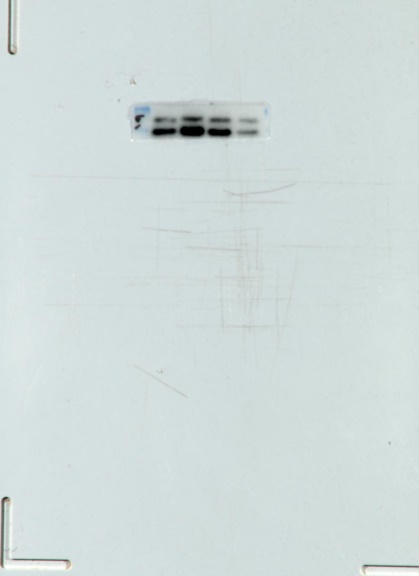

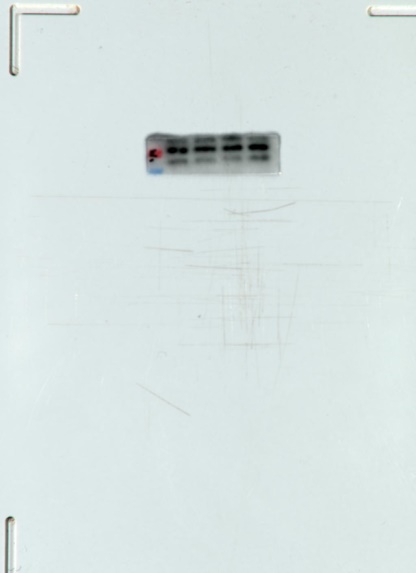

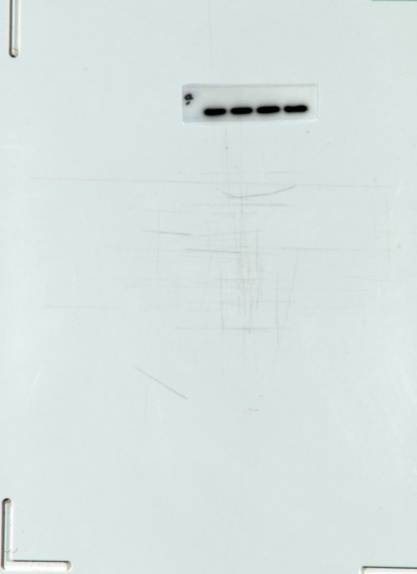


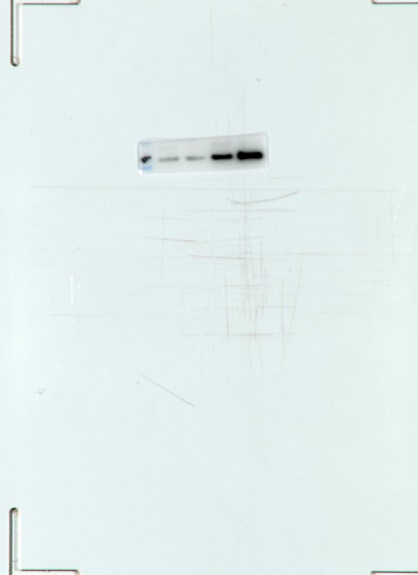

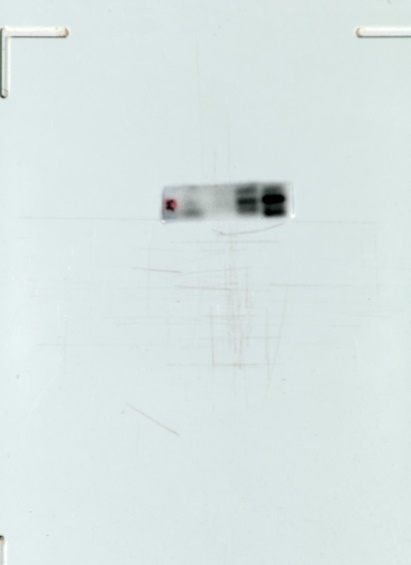

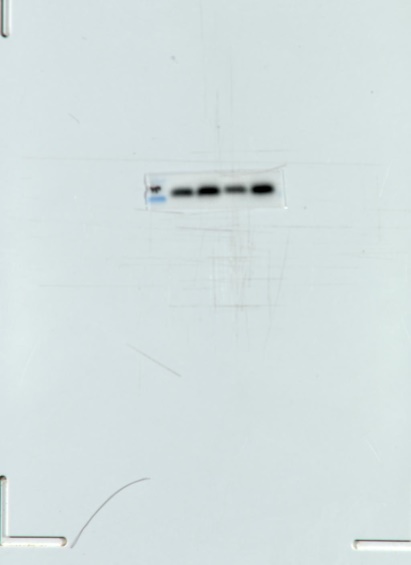

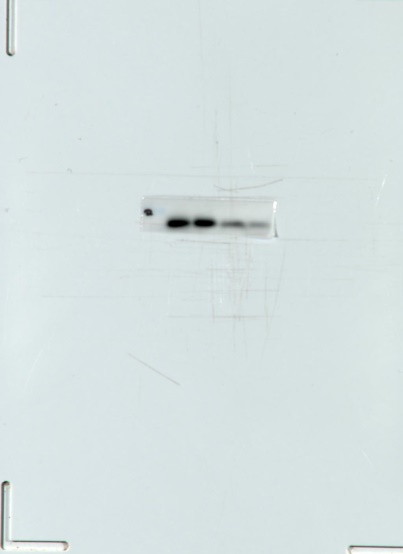

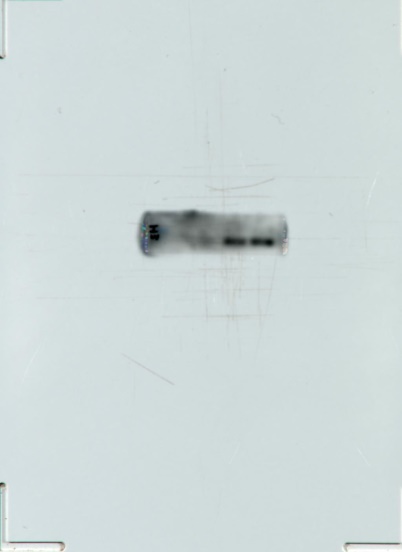


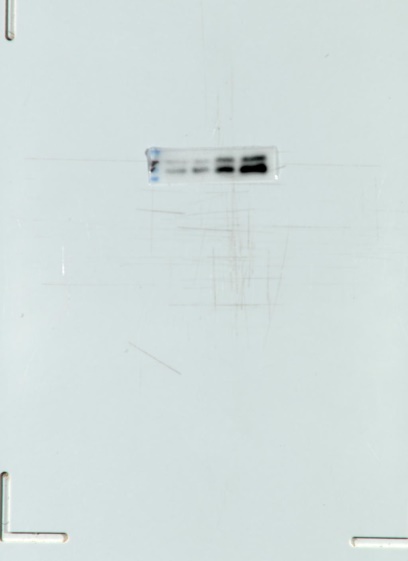

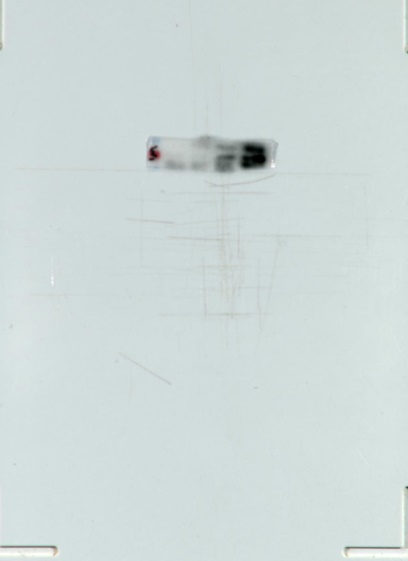

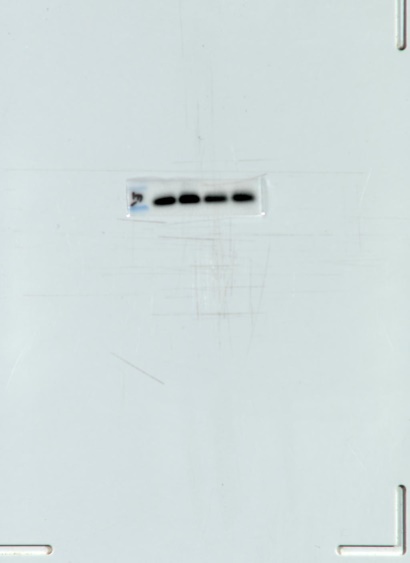


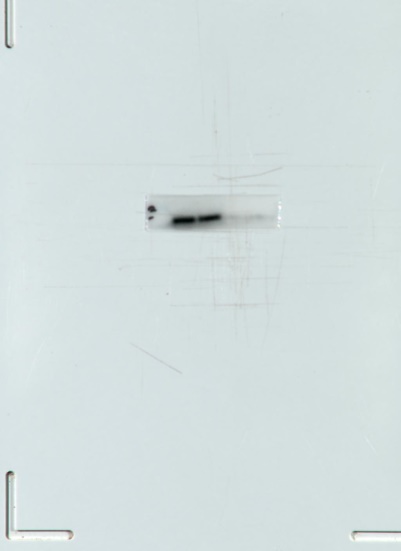

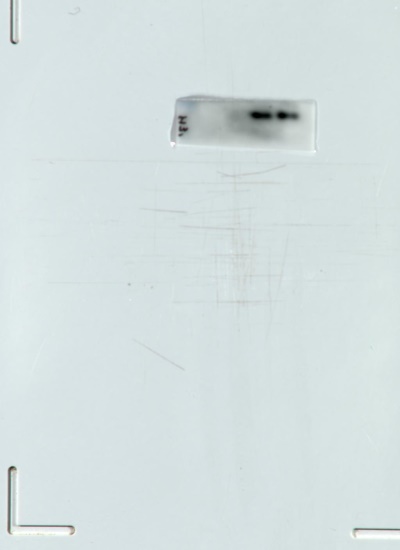


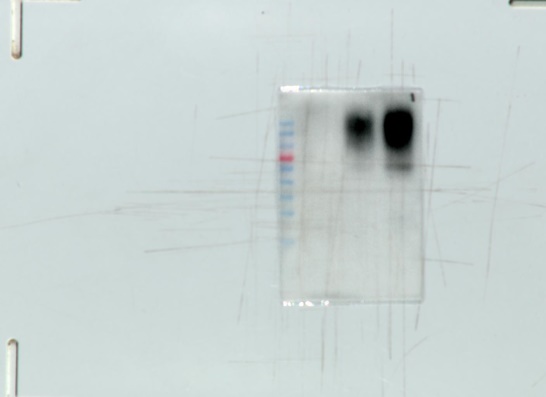

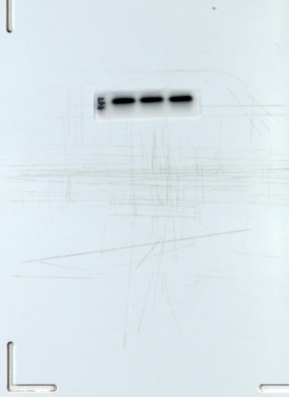

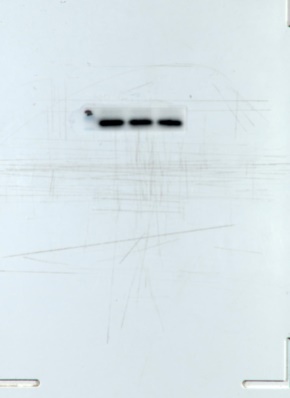


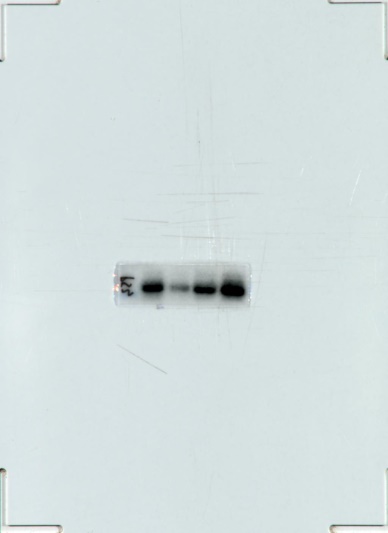

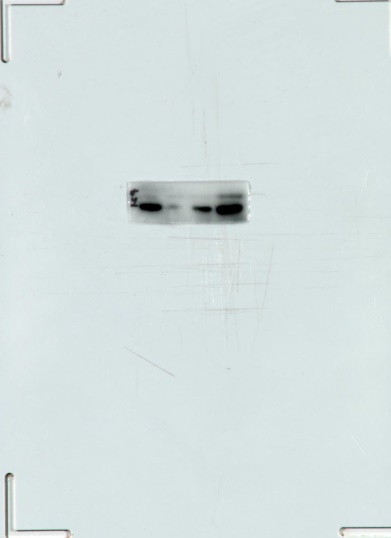

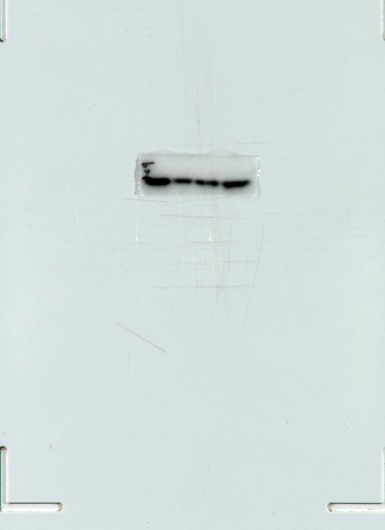


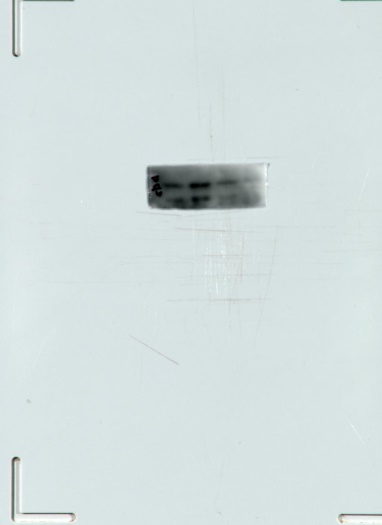

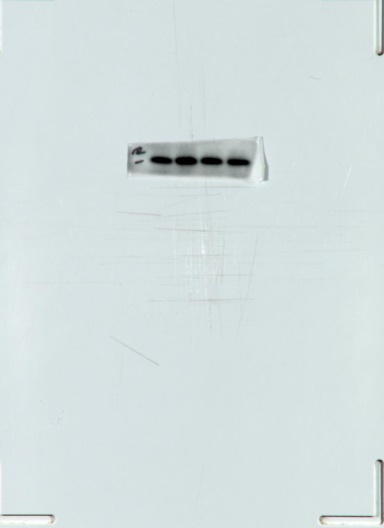


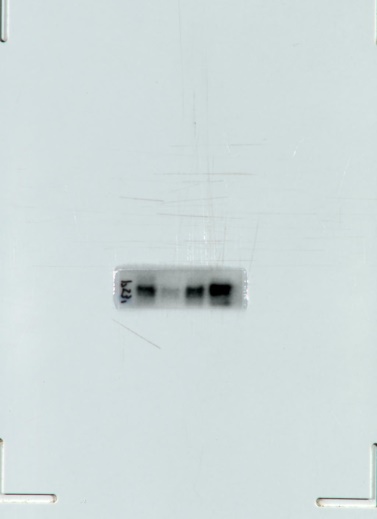

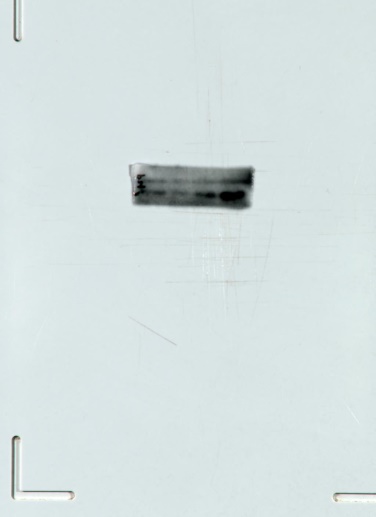

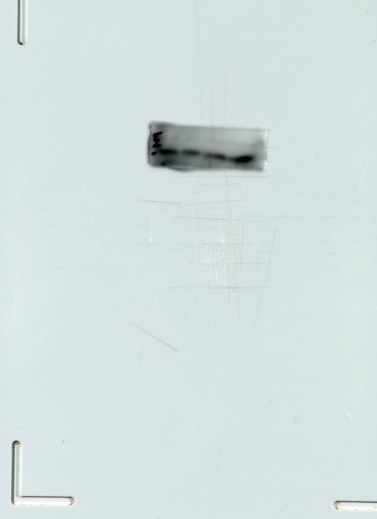

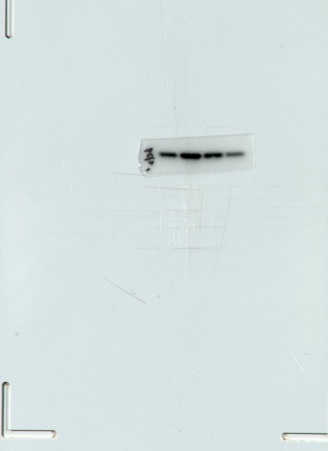

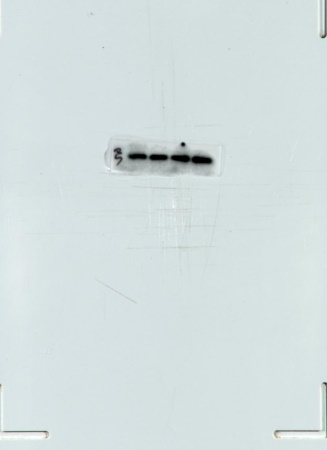


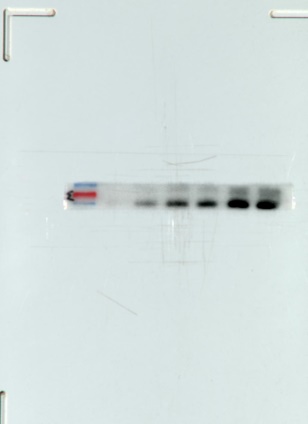

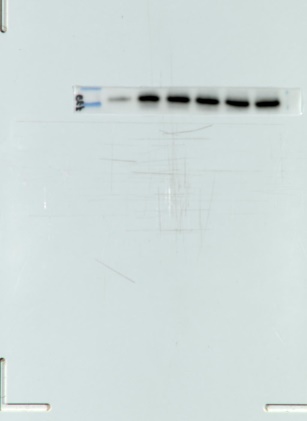

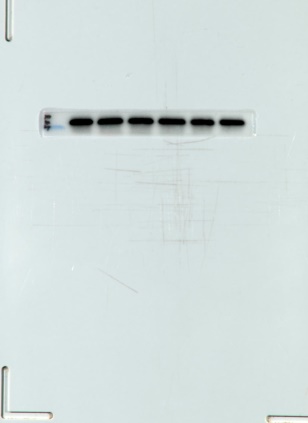

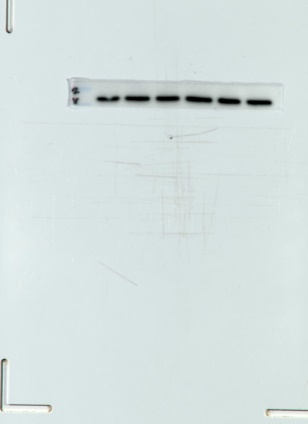


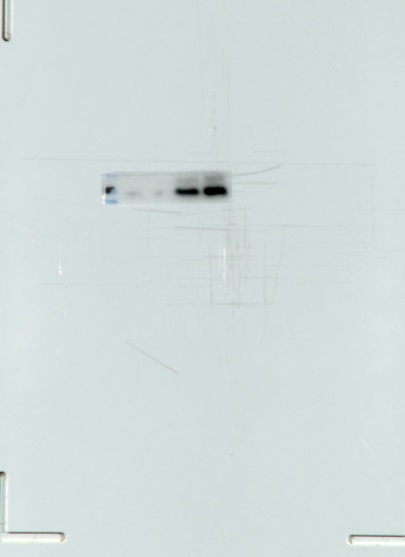

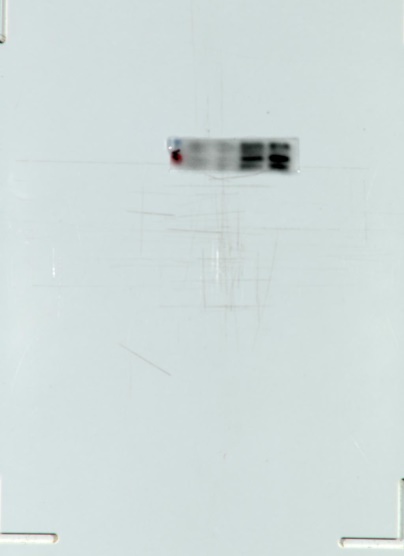

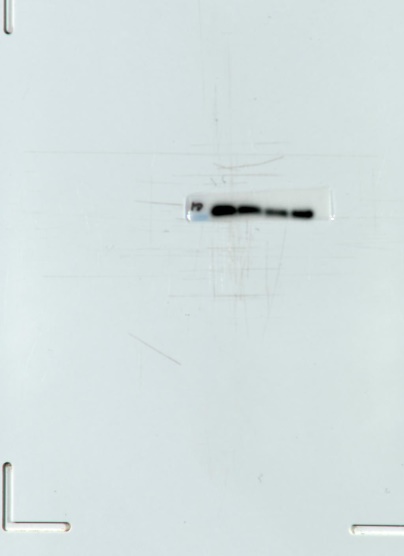

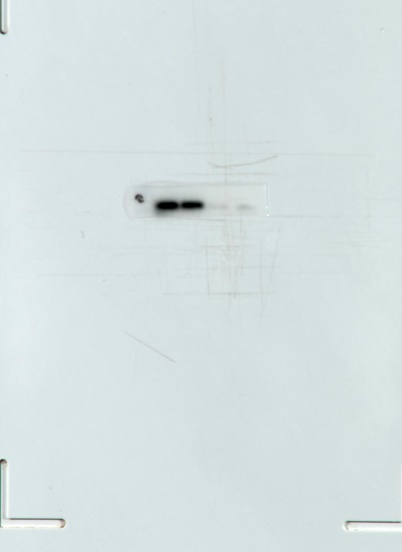

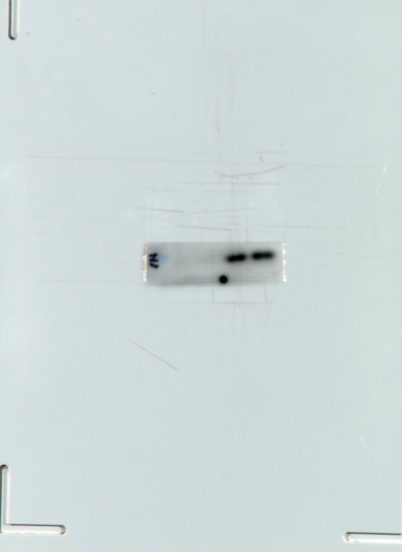


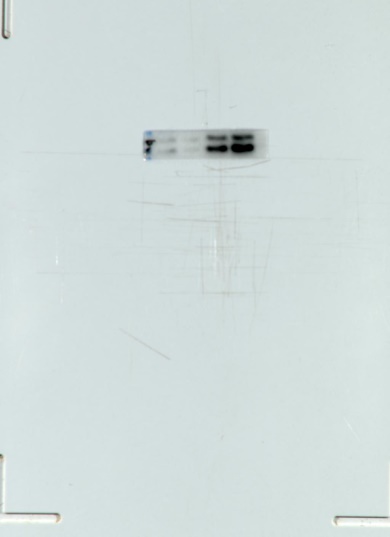

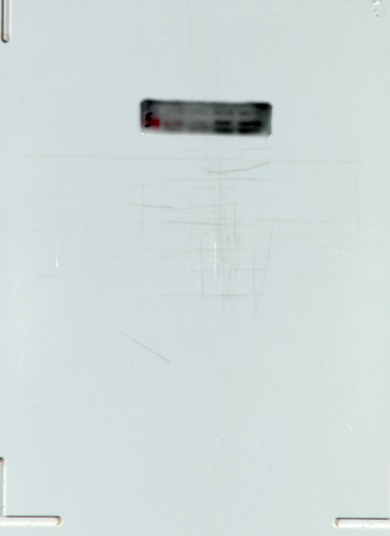

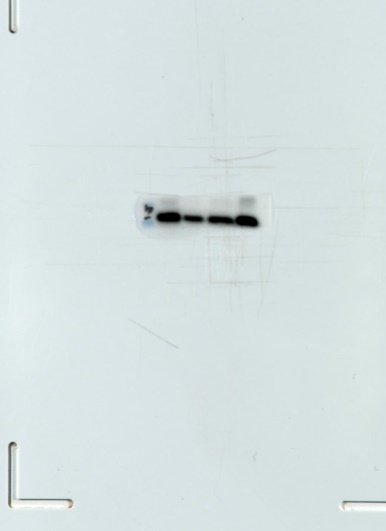

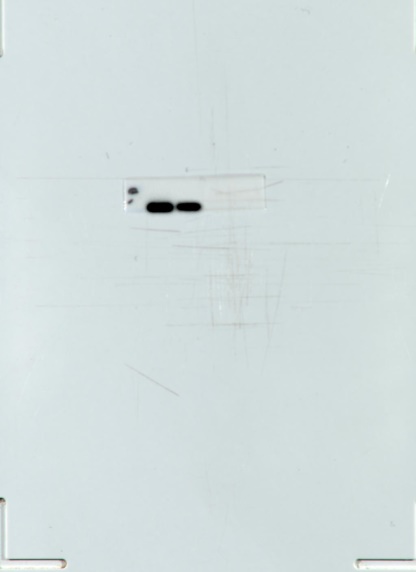

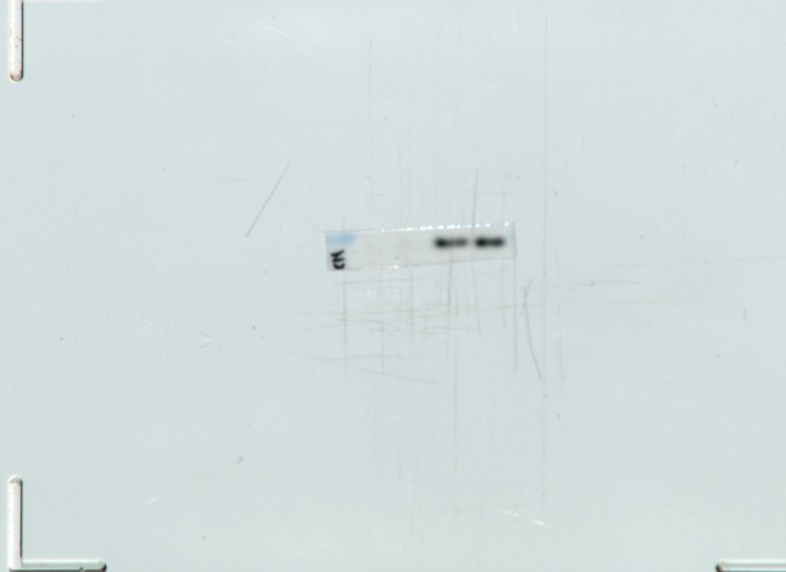


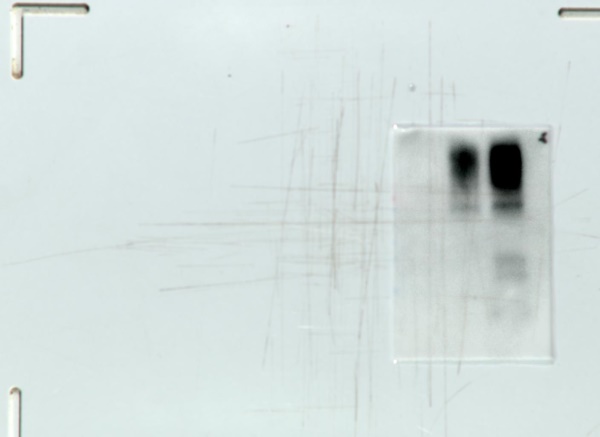

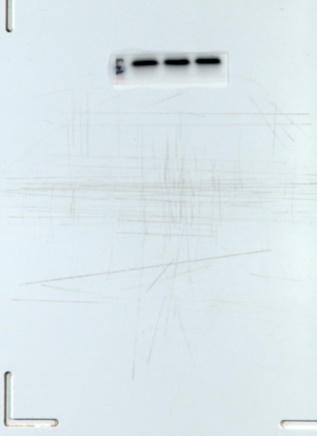

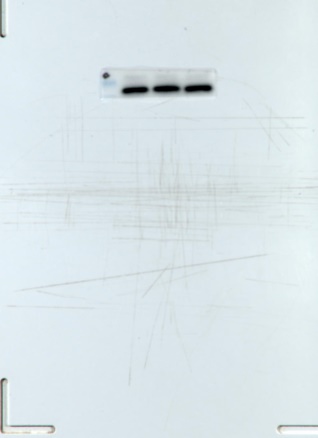


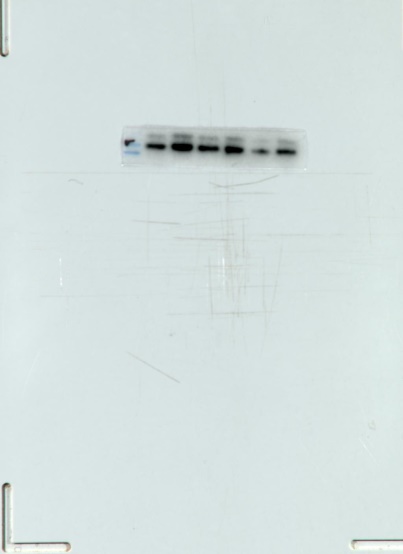

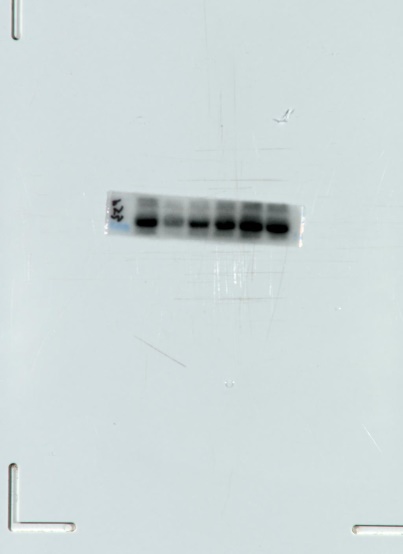

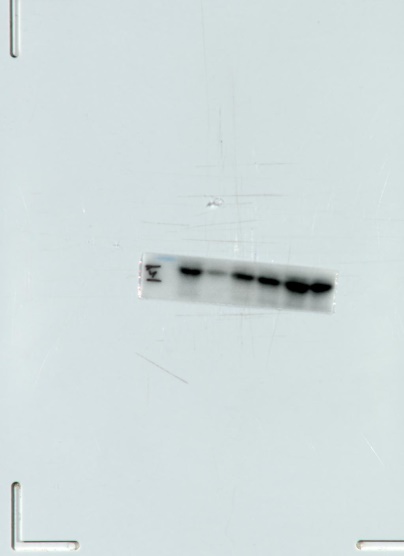

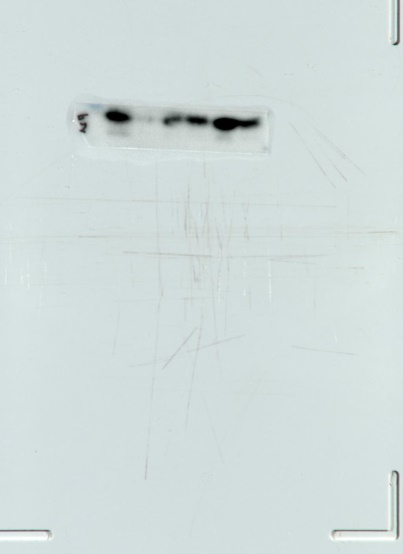

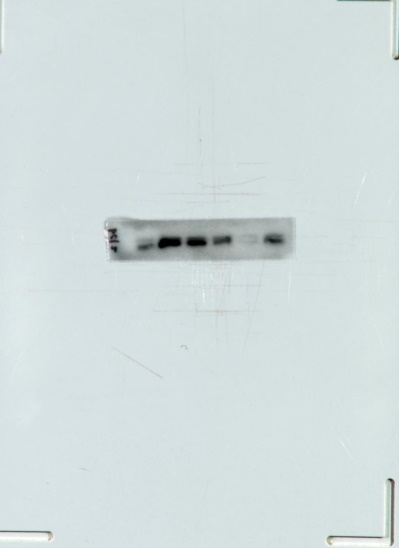

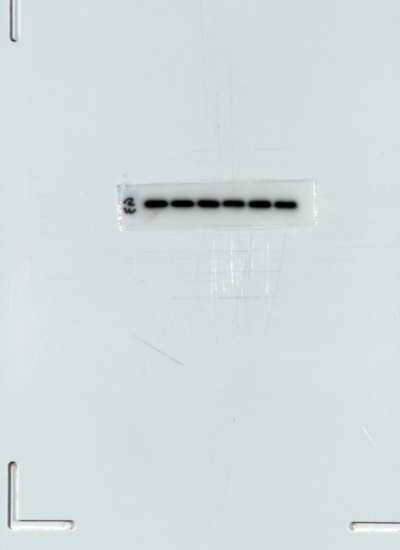


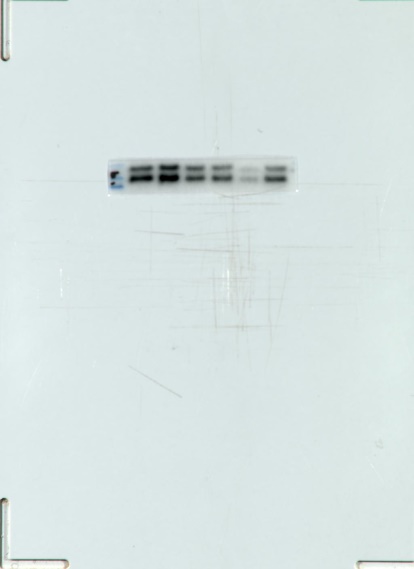

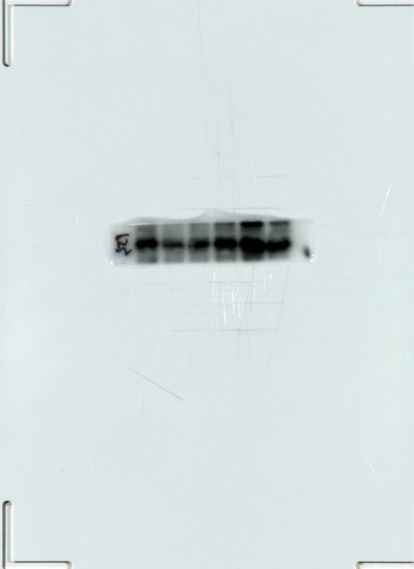

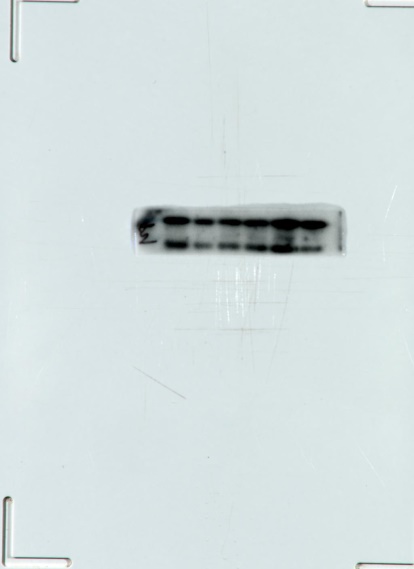

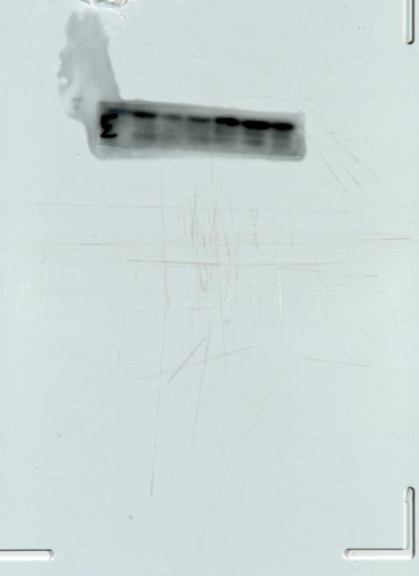

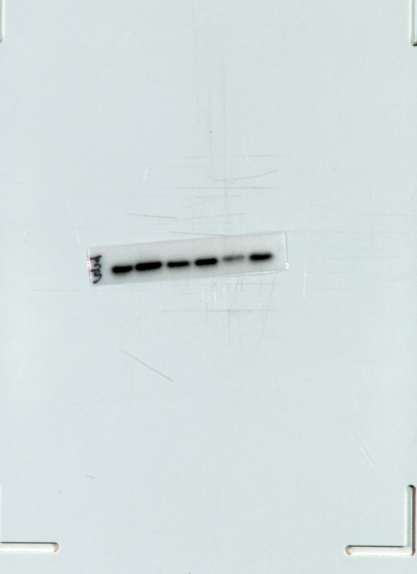

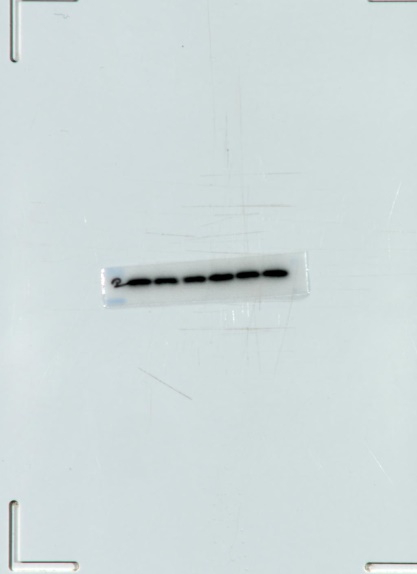


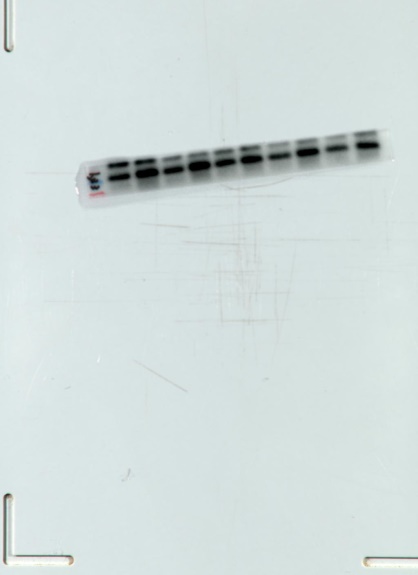

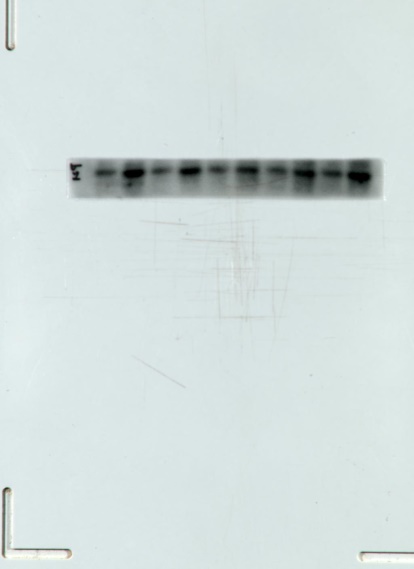

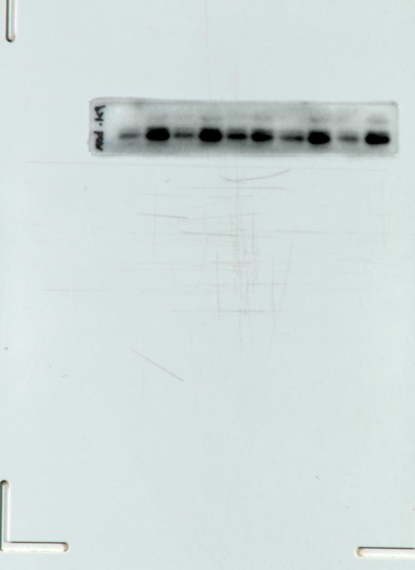

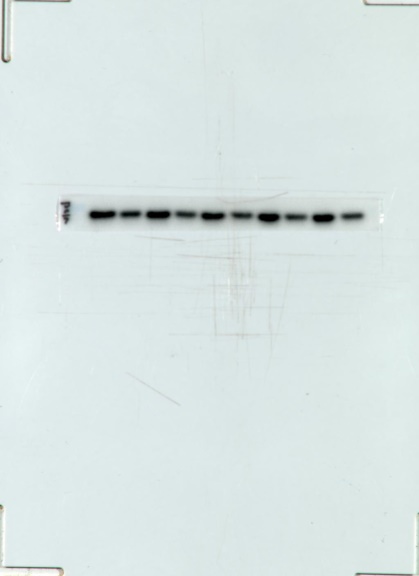

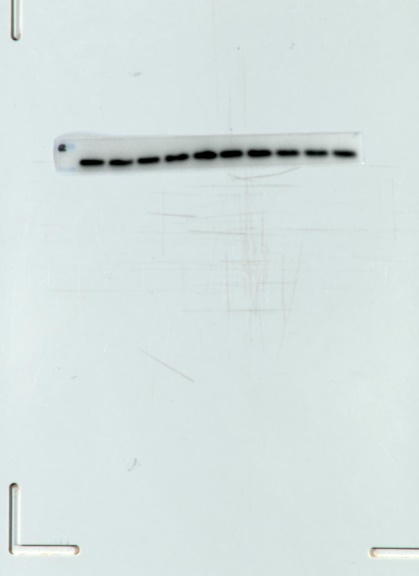


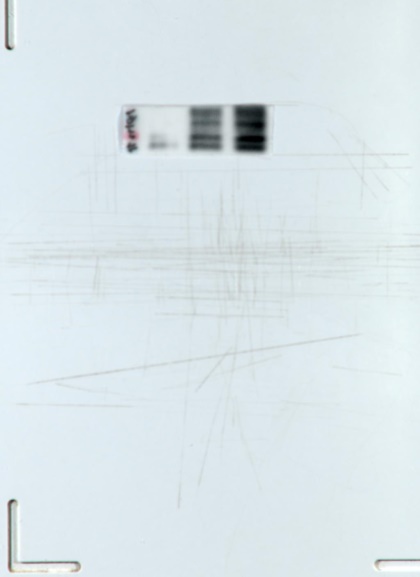

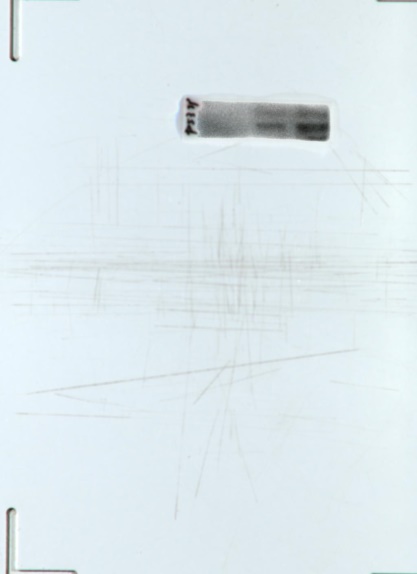

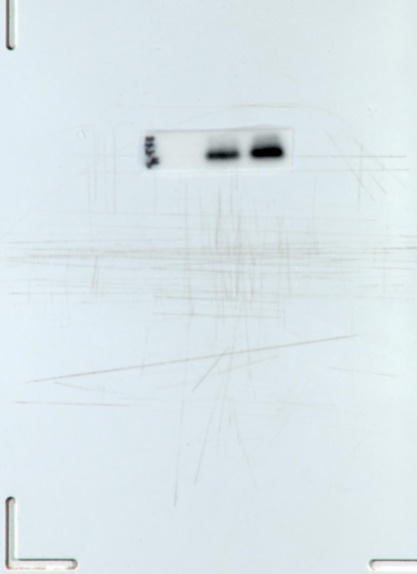

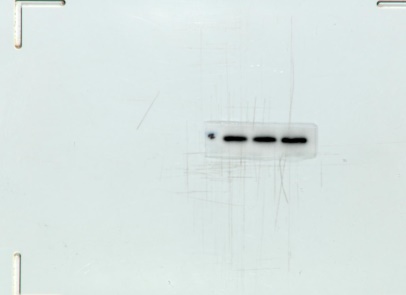


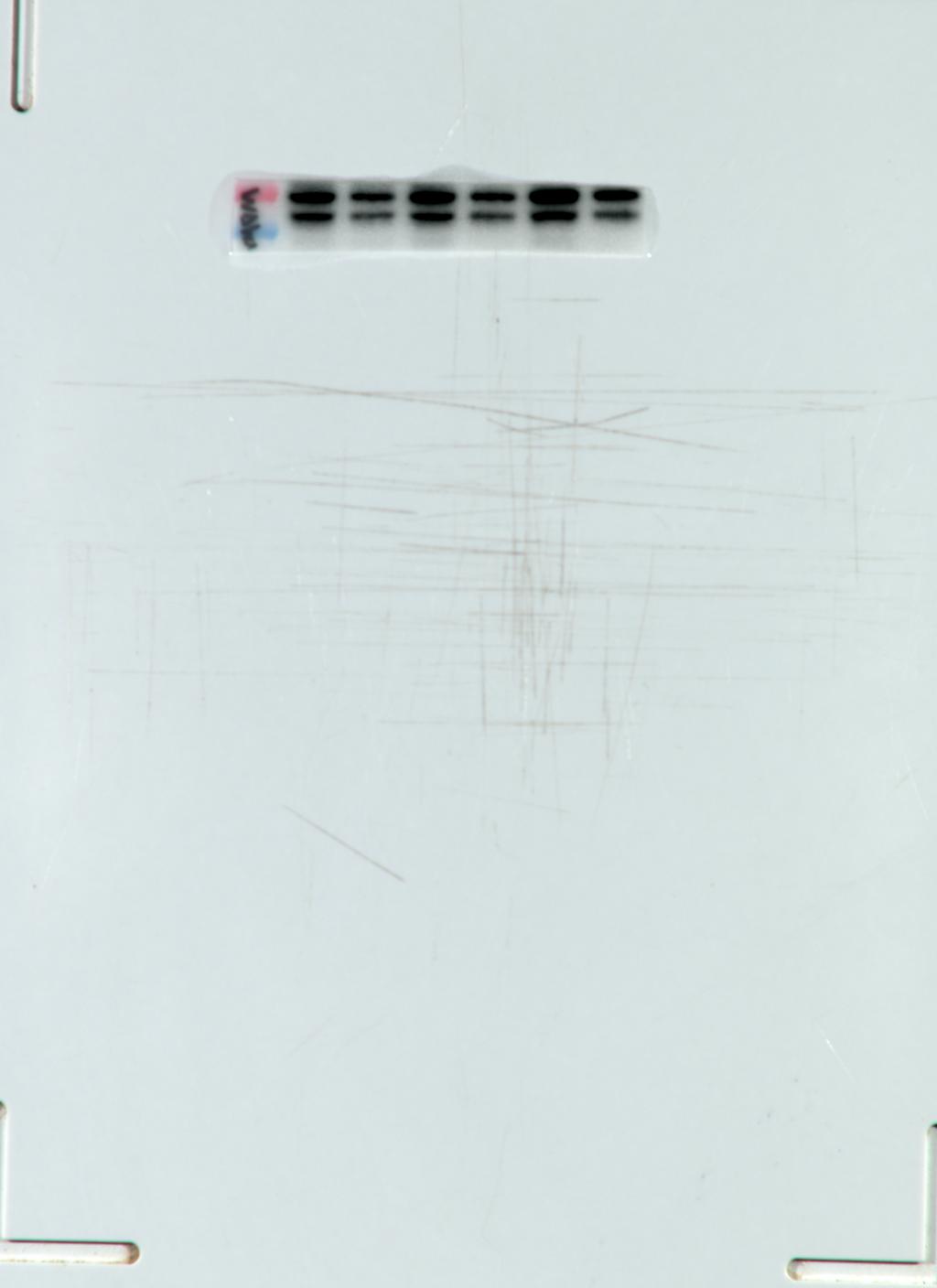

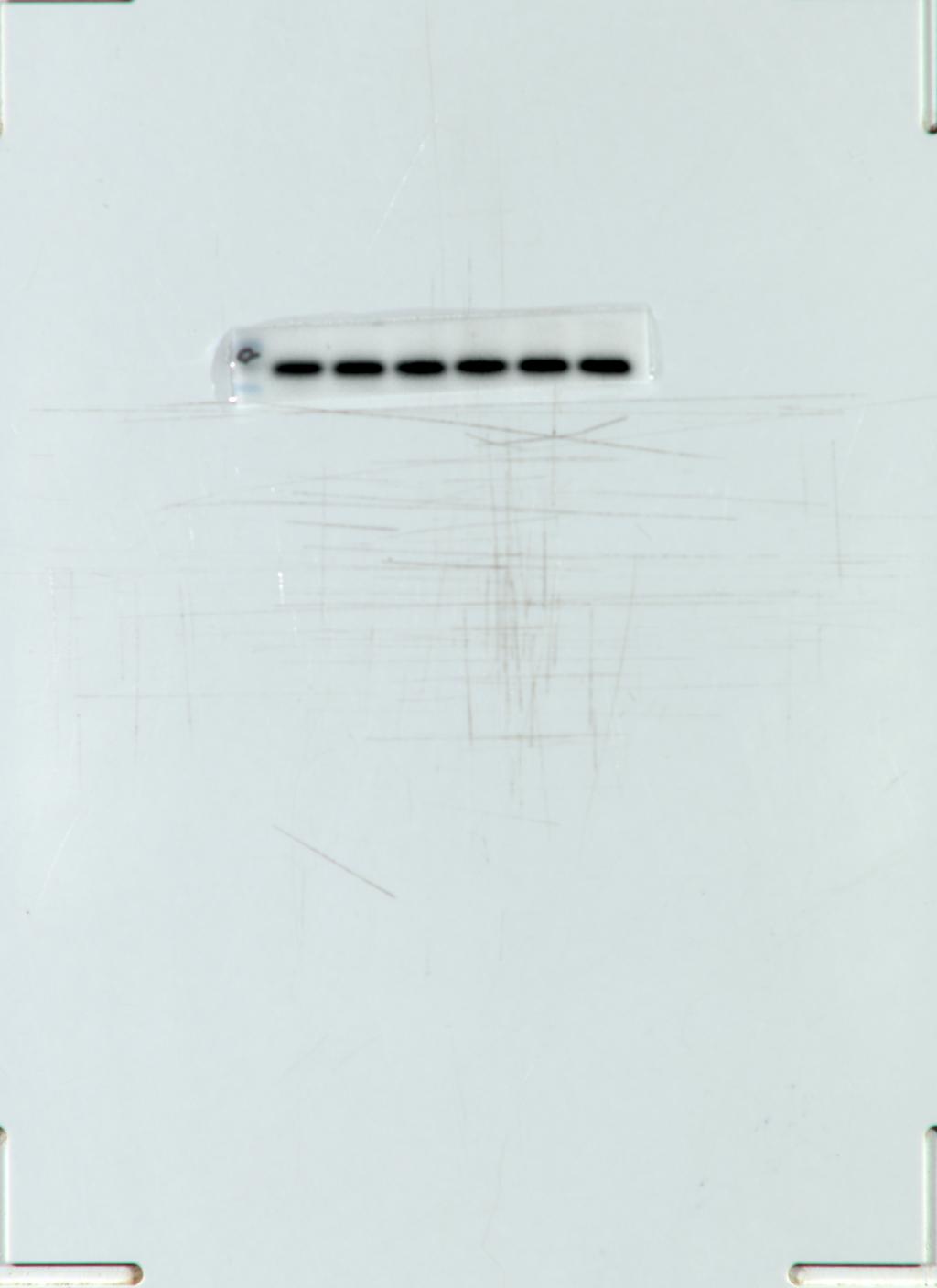

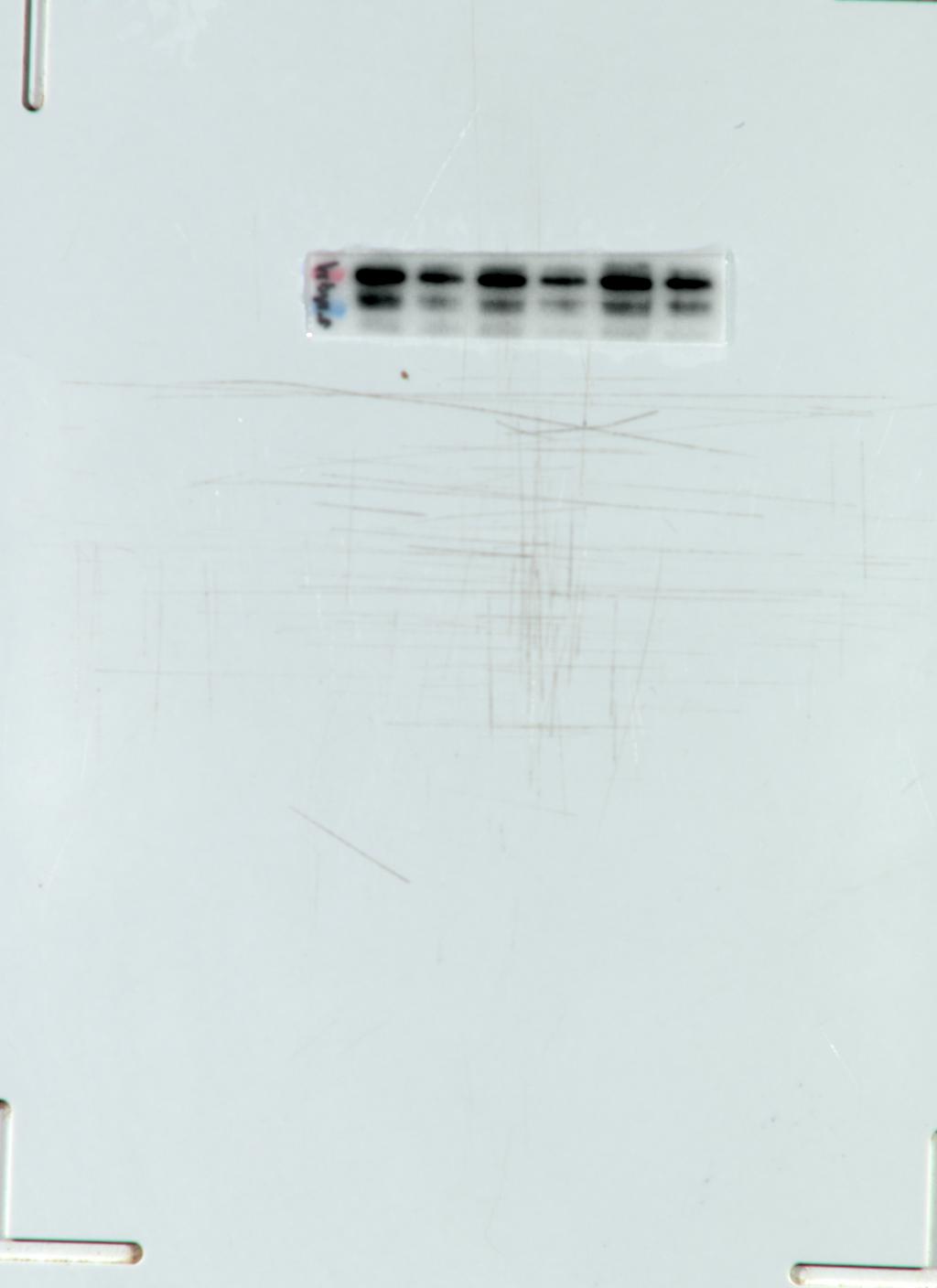

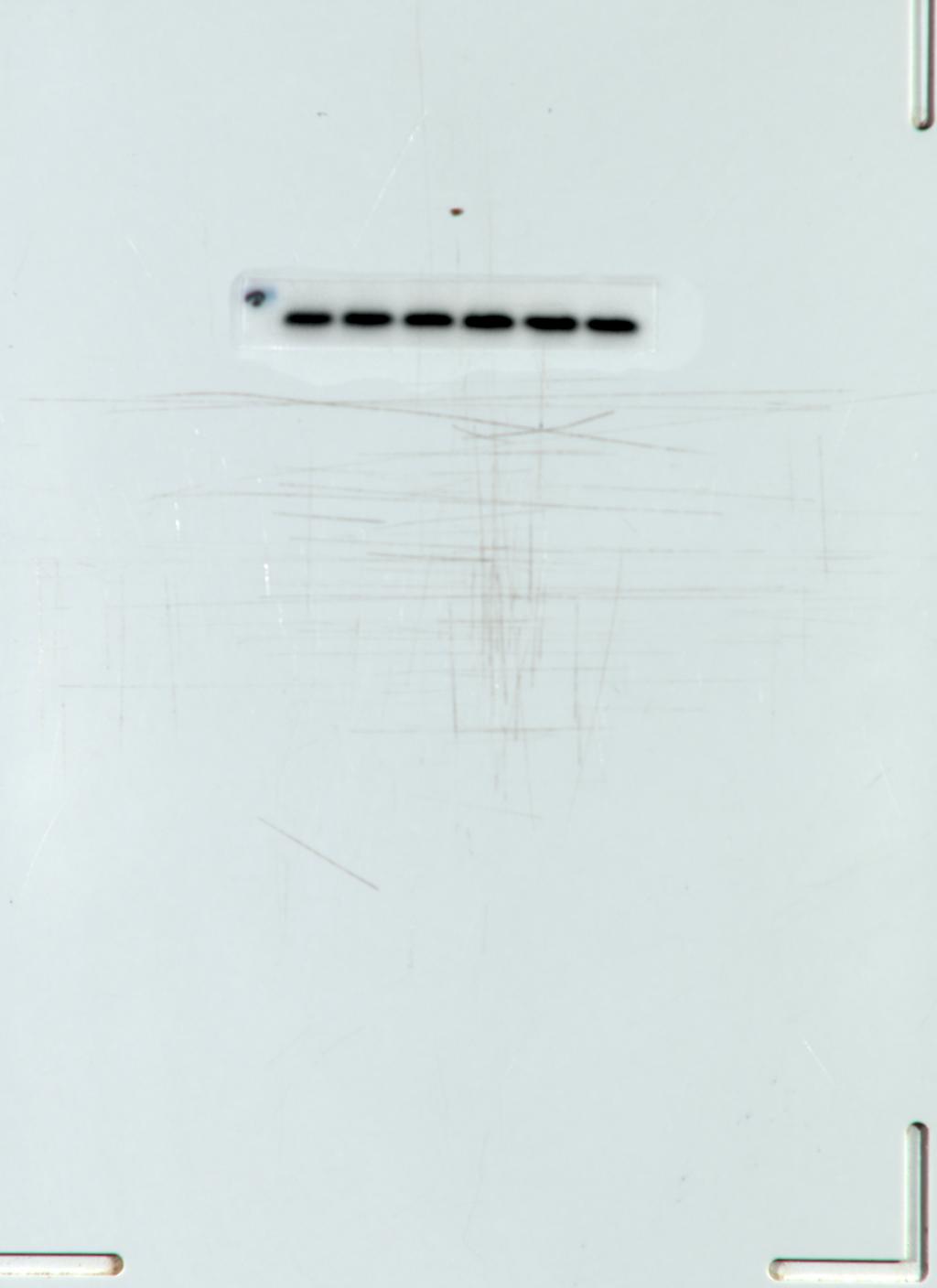

Supplement: Supplementary file 10 — Original Data File [file 41419_2022_4930_MOESM10_ESM.docx]
