# Supplementary material for: A novel 3’tRNA-derived fragment tRF-Val promotes proliferation and inhibits apoptosis by targeting EEF1A1 in gastric cancer
Source: Cell Death Dis. 2022 May 18;13(5):471. doi: 10.1038/s41419-022-04930-6 (PMC9117658; doi:10.1038/s41419-022-04930-6)
Supplement: Supplementary file 6 — Supplementary Figure Legends [file 41419_2022_4930_MOESM6_ESM.docx]

**Supplymentory Figure Legends**

**Supplymentory Fig. 1**

Regulatory relationships between tRF-Val and EEF1A1 are shown.

**A**. qRT-PCR assay indicated that tRF-Val overexpression and knockdown had no significant effect on the EEF1A1 mRNA expression in AGS and MKN-45 cells. **B**. The knockdown and overexpression efficiencies of EEF1A1 in AGS and MKN-45 cells were verified by WB. **C**. qRT-PCR assay indicated that EEF1A1 overexpression and knockdown had no significant effect on the tRF-Val expression in AGS and MKN-45 cells. **D**. WB assay indicated that tRF-Val overexpression and knockdown did not significantly affect the total EEF1A1 protein expression, but may promote its transport from cytoplasm to nucleus. **E**. Colony formation rescue assay indicated that the growth-promoting effect of tRF-Val overexpression on GC cells AGS and MKN-45 could be reversed by the knockdown of EEF1A1. **F**. EEF1A1 expression level was associated with poor prognosis in GC based on the Cancer Genome Atlas (TCGA) database ([www.kmplot.com](http://www.kmplot.com)). Data were shown as mean ± SD. (Student’s *t*-test, ***P* < 0.01 and ****P* < 0.001). n.s means no significance.

**Supplymentory Fig. 2**

EEF1A1 enhances the function of interacting proteins by acting as molecular chaperones.

**Supplymentory Fig. 3**

Co-IP assay was performed to detect the combination of EEF1A1 and MDM2-p53 complex in MKN-45 cells, and more MDM2-p53 was co-immunoprecipitated by anti-EEF1A1 antibody in the pcDNA EEF1A1 lane compared with pcDNA lane.

**Supplymentory Fig. 4**

tRF-Val promotes the proliferation of GC cells by regulating MDM2.

**A**. The knockdown efficiencies of MDM2 in AGS and MKN-45 cells were verified by WB. **B**, **C**. CCK-8 rescue assays indicated that the proliferation promoting effect of tRF-Val overexpression on GC cells AGS and MKN-45 could be reversed by the knockdown of MDM2. **D**, **E**. Colony formation rescue assays indicated that the growth-promoting effect of tRF-Val overexpression on GC cells AGS and MKN-45 could be reversed by the knockdown of MDM2. Data were shown as mean ± SD. (Student’s *t*-test, ***P* < 0.01 and ****P* < 0.001).

**Supplymentory Fig. 5**

Knockdown of tRF-Val inhibits the growth of MKN-45 cells in vivo.

**A**. The tumors of nude mice were dissected at 4 weeks after MKN-45 cells injection in sh-NC and sh-Val groups (*n* = 5 mice per group). **B**, **C**. The weights and volumes of the tumors were significantly decreased in the sh-Val group than those in the sh-NC group. Data were shown as mean ± SD. (Student’s *t*-test, ****P* < 0.001).
