# Supplementary material for: A novel 3’tRNA-derived fragment tRF-Val promotes proliferation and inhibits apoptosis by targeting EEF1A1 in gastric cancer
Source: Cell Death Dis. 2022 May 18;13(5):471. doi: 10.1038/s41419-022-04930-6 (PMC9117658; doi:10.1038/s41419-022-04930-6)
Supplement: Supplementary file 7 — The primer sequences of the genes in this experiment [file 41419_2022_4930_MOESM7_ESM.docx]

Supplementary Table 1:

The primer sequences of the genes in this experiment

| Genes | Primer sequence |
| --- | --- |
| β-actin | F:CATGTACGTTGCTATCCAGGC |
|  | R:CTCCTTAATGTCACGCACGAT |
| U6 | F: GGAACGATACAGAGAAGATTAGC |
|  | R: TGGAACGCTTCACGAATTTGCG |
| EEF1A1 | F: AAGGATGTTCGTCGTGGCAA |
|  | R: GCCGTGTGGCAATCCAATAC |
| tRF-58:75-Ala-AGC-1 | (5’-3’) GACTCCCCAGTACCTCCACCA |
| tRF-69:86-Leu-CAG-1 | (5’-3’) GCACATCCCACTCCTGACACC |
| tRF-60:76-Val-CAC-2 | (5’-3’) ACCGGGCAGAAGCACCAA |
| tRF-59:75-Gln-TTG-1-M3 | (5’-3’) GTGTCTCGGTGGGACCTCCA |
| tRF-60:76-Tyr-GTA-3-M2 | (5’-3’) CCGGCTCGGAGGACCAA |
