# Supplementary material for: A novel 3’tRNA-derived fragment tRF-Val promotes proliferation and inhibits apoptosis by targeting EEF1A1 in gastric cancer
Source: Cell Death Dis. 2022 May 18;13(5):471. doi: 10.1038/s41419-022-04930-6 (PMC9117658; doi:10.1038/s41419-022-04930-6)
Supplement: Supplementary file 8 — Supplementary Table 2 [file 41419_2022_4930_MOESM8_ESM.docx]

Supplementary Table 2:

Oligonucleotide sequences used in the cell transfection

| Groups | Oligonucleotide sequences |
| --- | --- |
| sh-Val | UGGUGCUUCUGCCCGGU |
| si-EEF1A1  si-MDM2 | CUACAAAAUUGGUGGUAUUTT  CUCUCGACUCAGAAGAUUAUA |
| si/sh-NC | UUCUCCGAACGUGUCACGU |
