# Supplementary material for: A novel 3’tRNA-derived fragment tRF-Val promotes proliferation and inhibits apoptosis by targeting EEF1A1 in gastric cancer
Source: Cell Death Dis. 2022 May 18;13(5):471. doi: 10.1038/s41419-022-04930-6 (PMC9117658; doi:10.1038/s41419-022-04930-6)
Supplement: Supplementary file 9 — The 37 specific tRF-Val binding proteins identified by mass spectrometry are shown. [file 41419_2022_4930_MOESM9_ESM.docx]

Supplementary Table 3:

The 37 specific tRF-Val binding proteins identified by mass spectrometry are shown.

| Accession | Genes | Coverage (%) | PSMs | Unique Peptides | Molecular Weight (kDa) | calc. pI |
| --- | --- | --- | --- | --- | --- | --- |
| P68104 | EEF1A1 | 14.07 | 25 | 6 | 50.1 | 9.01 |
| P05109 | S100A8 | 45.16 | 24 | 5 | 10.8 | 7.03 |
| P26373 | RPL13 | 27.49 | 24 | 7 | 24.2 | 11.65 |
| P14866 | HNRNPL | 21.39 | 21 | 9 | 64.1 | 8.22 |
| P06702 | S100A9 | 35.96 | 15 | 5 | 13.2 | 6.13 |
| P47929 | LGALS7 | 18.38 | 14 | 2 | 15.1 | 7.62 |
| Q08170 | SRSF4 | 5.06 | 14 | 1 | 56.6 | 11.52 |
| P06733 | ENO1 | 11.29 | 12 | 4 | 47.1 | 7.39 |
| Q13310 | PABPC4 | 6.21 | 11 | 1 | 70.7 | 9.26 |
| P38159 | RBMX | 11.00 | 11 | 4 | 42.3 | 10.05 |
| Q07020 | RPL18 | 18.09 | 10 | 3 | 21.6 | 11.72 |
| P07910 | HNRNPC | 16.34 | 10 | 4 | 33.6 | 5.08 |
| P67809 | YBX1 | 16.67 | 9 | 4 | 35.9 | 9.88 |
| P08865 | RPSA | 8.47 | 8 | 2 | 32.8 | 4.87 |
| P50454 | SERPINH1 | 6.46 | 8 | 2 | 46.4 | 8.69 |
| P02545 | LMNA | 4.97 | 8 | 3 | 74.1 | 7.02 |
| Q15517 | CDSN | 5.48 | 7 | 2 | 51.5 | 8.35 |
| P62995 | TRA2B | 6.94 | 7 | 2 | 33.6 | 11.25 |
| P62244 | RPS15A | 25.38 | 7 | 3 | 14.8 | 10.13 |
| P84098 | RPL19 | 13.27 | 6 | 2 | 23.5 | 11.47 |
| P60866 | RPS20 | 22.69 | 6 | 2 | 13.4 | 9.94 |
| P46781 | RPS9 | 8.25 | 6 | 2 | 22.6 | 10.65 |
| P42766 | RPL35 | 15.45 | 6 | 3 | 14.5 | 11.05 |
| A8K9J7 | Histone H2B | 19.05 | 5 | 2 | 14.0 | 10.32 |
| P11166 | SLC2A1 | 7.11 | 5 | 3 | 54.0 | 8.72 |
| P14678 | SNRPB | 12.08 | 5 | 2 | 24.6 | 11.19 |
| P40227 | CCT6A | 5.27 | 5 | 2 | 58.0 | 6.68 |
| O00571 | DDX3X | 3.78 | 4 | 2 | 73.2 | 7.18 |
| H0Y9R4 | RPL9 | 12.09 | 3 | 2 | 10.1 | 7.12 |
| B4DZC3 | cDNA FLJ55645 | 3.24 | 3 | 2 | 102.4 | 7.64 |
| K7EQH4 | ATP5A1 | 23.42 | 3 | 2 | 11.7 | 4.59 |
| P18077 | RPL35A | 18.18 | 3 | 2 | 12.5 | 11.06 |
| P18124 | RPL7 | 6.85 | 3 | 2 | 29.2 | 10.65 |
| O75223 | GGCT | 13.30 | 3 | 2 | 21.0 | 5.14 |
| P62258 | YWHAE | 7.45 | 2 | 2 | 29.2 | 4.74 |
| P06576 | ATP5B | 5.67 | 2 | 2 | 56.5 | 5.40 |
| P31943 | HNRNPH1 | 4.90 | 2 | 2 | 49.2 | 6.30 |
