# Supplementary material for: A novel 3’tRNA-derived fragment tRF-Val promotes proliferation and inhibits apoptosis by targeting EEF1A1 in gastric cancer
Source: Cell Death Dis. 2022 May 18;13(5):471. doi: 10.1038/s41419-022-04930-6 (PMC9117658; doi:10.1038/s41419-022-04930-6)
Supplement: Supplementary file 12 — cdd-author-contribution-form [file 41419_2022_4930_MOESM12_ESM.pdf]

DECLARATION OF CONTRIBUTIONS TO ARTICLE

ADMC

Manuscript Number: CDD-22-2944

Journal Name: *Cell Death & Differentiation* (the 'Journal')

Proposed Title of the Contribution:

A novel 3'tRNA-derived fragment tRF-Val promotes proliferation and inhibits apoptosis by targeting EEF1A1 in gastric cancer (the 'Contribution)

Author(s):

Huaiping Cui, Han Li, Hao Wu, Fengying Du, Xiaozhou Xie, Shujie Zeng, Zihao Zhang, Kangdi Dong, Liang Shang, Changqing Jing, Leping Li (the 'Authors')

For all *CDD* articles, each person named as an author in the published version must be able to show he or she has contributed substantially to the article.

Authorship credit should be based on 1) substantial contributions to conception and design, acquisition of data, or analysis and interpretation of data; 2) drafting the article or revising it critically for important intellectual content; and 3) final approval of the version to be published. Authors should meet conditions 1, 2 and 3.

Any person who cannot be shown to have made a substantial contribution to the article cannot be listed as an author in the final version. The name of any person who is deemed to have made a minor contribution can, however, appear in the Acknowledgments section of the article.

Please complete the table below to indicate the contributions of all named authors to the manuscript.

Author Full Name:	Specification of Contribution to the Manuscript:
Huaiping Cui	H. C. performed development of methodology and writing, review and revision of the paper
Han Li	H. L. performed development of methodology and writing, review and revision of the paper
Hao Wu	H. W. performed development of methodology and writing, review and revision of the paper
Fengying Du	F. D. performed development of methodology and writing, review and revision of the paper
Xiaozhou Xie	X. X. provided acquisition, analysis and interpretation of data, and statistical analysis
Shujie Zeng	S. Z. provided acquisition, analysis and interpretation of data, and statistical analysis
Zihao Zhang	Z. Z. provided acquisition, analysis and interpretation of data, and statistical analysis
Kangdi Dong	K. D. provided acquisition, analysis and interpretation of data, and statistical analysis
Liang Shang	L. S. performed study concept and design
Changqing Jing	C. J. performed study concept and design
Leping Li	L. S. performed study concept and design

Please complete the table below to indicate the contributions of all named authors to the figures.

Figure 1:

In figure 1, L.L., L.S. and C.J. performed study concept and design.

Figure 2:

In figure 2, H.C. generated the tRFs data, H.L., H.W. and F.D. generated the PCR data

Figure 3:

In figure 3, H.C., X.X., S.Z., Z.Z. and K.D. performed the CCK-8 assay, colony formation assay, flow cytometry apoptosis detection, and transwell assay.

Figure 4:

In figure4, H.C. generated the RNA-pulldown assay, PCR and western data and assembled the figure. S. Z. generated the IF data.

Figure 5:

In figure 5, H.C. generated the Co-IP assay, colony formation assay, flow cytometry apoptosis detection. X.X., S.Z., Z.Z. and K.D generated the western data and assembled the figure.

Figure 6:

In figure 6, H. L. generated the Co-IP assay and IF assay data and assembled the figure. X.X., S.Z., Z.Z. and K.D generated the western data and assembled the figure.

In figure 7, H. W. performed the animal experiments. H.C. generated the schematic diagram.

Signed for and on behalf of the Author(s):	Print Name:	Date:
[signature]	Leping Li	2022.1.6
